# Adipose tissue deficiency impairs transient lipid accumulation and delays liver regeneration following partial hepatectomy in male Seipin knockout mice

**DOI:** 10.1002/ctm2.70238

**Published:** 2025-02-20

**Authors:** Qianqian Dong, Ziwei Liu, Yidan Ma, Xin Chen, Xiaowei Wang, Jinye Tang, Kexin Ma, Chenxi Liang, Mengyu Wang, Xiaoqin Wu, Yang Liu, Yaru Zhou, Hongyuan Yang, Mingming Gao

**Affiliations:** ^1^ Department of Biochemistry and Molecular Biology The Key Laboratory of Neural and Vascular Biology Ministry of Education, The Key Laboratory of Vascular Biology of Hebei Province, Cardiovascular Medical Science Center, Hebei Medical University Shijiazhuang Hebei China; ^2^ Department of Clinical Laboratory The Second Hospital of Hebei Medical University Shijiazhuang Hebei China; ^3^ Department of Clinical Laboratory Bethune International Peace Hospital Shijiazhuang Hebei China; ^4^ Department of General Surgery The First Hospital of Hebei Medical University Shijiazhuang Hebei China; ^5^ Department of Cardiology First Affiliated Hospital of Zhengzhou University Zhengzhou Henan China; ^6^ Department of Integrative Biology and Pharmacology University of Texas Health Science Center at Houston Houston Texas USA; ^7^ Department of Endocrinology The Third Hospital of Hebei Medical University Shijiazhuang Hebei China

**Keywords:** adipose tissue transplantation, hepatocyte proliferation, liver regeneration, partial hepatectomy, Seipin knockout mice, transient regeneration‐associated steatosis (TRAS)

## Abstract

**Background:**

Liver diseases pose significant health challenges, underscoring the importance of understanding liver regeneration mechanisms. Systemic adipose tissue is thought to be a primary source of lipids and energy during this process; however, empirical data on the effects of adipose tissue deficiency are limited. This study investigates the role of adipose tissue in liver regeneration, focusing on transient regeneration‐associated steatosis (TRAS) and hepatocyte proliferation using a Seipin knockout mouse model that mimics severe human lipodystrophy. Additionally, the study explores therapeutic strategies through adipose tissue transplantation.

**Methods:**

Male Seipin knockout (*Seipin^−/−^*) and wild‐type (WT) mice underwent 2/3 partial hepatectomy (PHx). Liver and plasma samples were collected at various time points post‐surgery. Histological assessments, lipid accumulation analyses and measurements of hepatocyte proliferation markers were conducted. Additionally, normal adipose tissue was transplanted into *Seipin^−/−^* mice to evaluate the restoration of liver regeneration.

**Results:**

*Seipin^−/−^* mice exhibited significantly reduced liver regeneration rates and impaired TRAS, as evidenced by histological and lipid measurements. While WT mice demonstrated extensive hepatocyte proliferation at 48 and 72 h post‐PHx, characterised by increased mitotic cells, elevated proliferating cell nuclear antigen and Ki67 expression, *Seipin^−/−^* mice showed delayed hepatocyte proliferation. Notably, adipose tissue transplantation into *Seipin^−/−^* mice restored TRAS and improved liver regeneration and hepatocyte proliferation. Conversely, liver‐specific overexpression of Seipin in *Seipin^−/−^* mice did not affect TRAS or liver regeneration, indicating that the observed effects are primarily due to adipose tissue deficiency rather than hepatic Seipin itself.

**Conclusions:**

Systemic adipose tissue is essential for TRAS and effective liver regeneration following PHx. Its deficiency impairs these processes, while adipose tissue transplantation can restore normal liver function. These findings underscore the critical role of adipose tissue in liver recovery and suggest potential therapeutic strategies for liver diseases associated with lipodystrophies.

**Key points:**

- *Seipin^−/−^* mice, which lack adipose tissue, exhibit significantly impaired TRAS and delayed liver regeneration following partial hepatectomy.
- Transplantation of normal adipose tissue into *Seipin^−/−^* mice restores TRAS and enhances liver regeneration, highlighting the essential role of adipose tissue in these processes.
- Liver‐specific overexpression of Seipin has no effect on TRAS and liver regeneration in *Seipin^−/−^* mice.

## Graphical abstract

1. *Seipin^−/−^* mice, which lack adipose tissue, exhibit significantly impaired TRAS and delayed liver regeneration following partial hepatectomy.
2. Transplantation of normal adipose tissue into *Seipin^−/−^* mice restores TRAS and enhances liver regeneration, highlighting the essential role of adipose tissue in these processes.
3. Liver‐specific overexpression of Seipin has no effect on TRAS and liver regeneration in *Seipin^−/−^* mice.

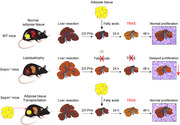

## 1 BACKGROUND

Liver conditions such as metabolic‐associated fatty liver disease, hepatitis and hepatocellular carcinoma pose significant threats to human health. The liver's ability to regenerate is crucial for recovery following partial hepatectomy (PHx) (e.g., tumour resection or living donor liver transplantation) and for healing from acute or chronic liver damage caused by infections, toxins, immune dysfunction, metabolic disorders or other factors. In rodents, PHx is regarded as a well‐established model for investigating liver regeneration, initiating regenerative processes within hours post‐surgery. After performing a typical two‐thirds PHx in rodents, most of the liver volume is replenished within 7–8 days, with full restoration accomplished within 3 weeks.^1^, ^2^, ^3^

During early liver regeneration, hepatocytes transiently accumulate substantial amounts of lipids, a phenomenon known as transient regeneration‐associated steatosis (TRAS). This lipid accumulation provides the energy necessary for cell proliferation.^4^, ^5^ Previous studies have demonstrated that following PHx, the regenerated liver relies heavily on fatty acid β‐oxidation for energy production.^6^, ^7^ Inhibition of β‐oxidation can impede liver regeneration,^8^ while lipid emulsions or carnitine, which enhance β‐oxidation, can accelerate liver regeneration post‐PHx.^9^ These findings underscore the critical role of β‐oxidation‐derived energy in liver regeneration. Although fatty acids for triglyceride synthesis in the liver can come from de novo synthesis or circulation, studies by Newberry et al.^10^ using liver‐specific fatty acid synthase knockout mice (L‐FAS‐KO) indicated that de novo synthesis inhibition did not affect liver triglyceride accumulation or regenerative capacity, suggesting that de novo synthesis might not be essential for fatty acids needed during liver regeneration. Adipose tissue, serving as a reservoir for triglycerides, rapidly mobilises and releases free fatty acids (FFAs) when required.^11^ In the context of early liver injury, the release of catecholamines, which promotes fat mobilisation, suggests that systemic adipose tissue may be a primary source for lipid accumulation and energy during liver regeneration.^3^, ^12^ Investigating the role of adipose tissue in TRAS post‐PHx and its impact on liver regeneration may provide novel insights for liver disease therapies.

Obesity, characterised by abnormal adipocyte enlargement and increased adipose tissue mass, impairs the responsiveness of adipocytes to lipolytic signals, leading to reduced fat utilisation. Studies in genetic obesity models, such as *ob/ob* or *db/db* mice, have shown impaired liver regeneration despite significant hepatic lipid accumulation,^13^, ^14^, ^15^, ^16^ indicating that dysfunctional adipose tissue in obesity may inhibit liver regeneration. Conversely, metabolic disorders resulting from partial or complete loss of adipose tissue, known as lipodystrophy, have been less studied regarding their effects on liver regeneration.

Seipin (BSCL2) is essential for maintaining both cellular and systemic lipid storage. At the cellular level, Seipin is crucial for the formation of lipid droplets, while at the systemic level, it is vital for adipogenesis.^17^, ^18^ Loss‐of‐function mutations in Seipin cause congenital generalized lipodystrophy type 2 (CGL2), characterised by severe loss of adipose tissue, hypertriglyceridemia, fatty liver and insulin resistance.^19^ Seipin knockout mice (*Seipin^−/−^*) exhibit phenotypes consistent with CGL2 patients.^20^, ^21^ Although Seipin deficiency results in severe fatty liver, Seipin is minimally expressed in hepatocytes. Previous studies have shown that liver‐specific Seipin knockout mice do not display significant liver abnormalities,^22^, ^23^ while transplantation of normal adipose tissue can rescue the fatty liver phenotype in *Seipin^−/−^* mice.^24^, ^25^ This suggests that liver disease in *Seipin^−/−^* mice is due to the absence of adipose tissue rather than the lack of Seipin in the liver. Therefore, *Seipin^−/−^* mice serve as an excellent model for studying the impact of adipose tissue loss on liver function. However, the role of Seipin deficiency‐induced adipose tissue loss in liver regeneration remains unexplored.

In this study, we utilised *Seipin^−/−^* mice as a model of systemic adipose tissue deficiency to investigate the role of adipose tissue in post‐PHx transient lipid deposition and liver regeneration. We found that Seipin deficiency impairs TRAS and delays hepatocyte proliferation, while transplantation of normal adipose tissue can restore these processes. Our findings emphasise the critical role of adipose tissue in liver regeneration following hepatectomy.

## 2 MATERIALS AND METHODS

### 2.1 Animal

The *Seipin* knockout mouse was constructed using CRISPR/Cas9 system, as described previously.^26^ *Seipin* heterozygous (*Seipin* ^+/−^) male and female mice were bred to generate *Seipin* homozygous knockout (*Seipin^−/−^*) mice and littermate wild‐type (WT) control mice. Male mice were used in this study. All animals were housed in the Animal Center of Hebei Medical University under barrier system conditions, with controlled environmental parameters: room temperature maintained between 22 and 26°C, relative humidity between 40 and 60%, and a 12:12 h light/dark cycle. They had ad libitum access to food and water. The experimental design and protocols were approved by the Laboratory Animal Ethical and Welfare Committee of Hebei Medical University (IACUC‐Hebmu‐P 2023031).

### 2.2 Partial hepatectomy

*Seipin^−/−^* and littermate WT male mice, aged 11–12 weeks, were used in this study. The two‐thirds PHx surgery was performed following a previously established method.^27^ Prior to surgery, mice were anaesthetised by an intraperitoneal injection of 1% sodium pentobarbital, administered at 7 µL per gram of body weight. A midline abdominal incision, approximately 3 cm long, was made through the skin and muscle to expose the xiphoid process. *First resection*: Four‐0 sutures were placed at the root of the left lobule of the liver, with at least two surgical knots made at the root. The ligated left lobe was then resected once it turned dark red. *Second resection*: Four‐0 sutures were placed between the stump and the median lobe. The median lobe was pulled down over the suture, with the knot placed above the gallbladder (which would be removed) but no closer than 2 mm from the suprahepatic vena cava. Three surgical knots were made. As the median lobe turned dark red, the dark red portion outside the knot was resected. *Suture*: The peritoneum and skin were closed after confirming there was no bleeding in the abdominal cavity.

*Calculation of liver regeneration rate (LRR)*: Liver weight recovery was assessed as the percentage of the regenerated liver mass, as described previously.^14^ The LRR was determined using the formula: LRR (%) = 100 × (*C* − (A − *B*))/*A*, where *A* represents the estimated total liver weight at the time of resection (= *B *× 100/65), *B* is the weight of the liver excised, and *C* is the weight of the regenerated liver at the time of sacrifice.

### 2.3 Adipose tissue transplantation

The adipose tissue transplantation (AT) surgery was performed as previously described.^28^ In this study, *Seipin^−/−^* mice aged 8–10 weeks were used as recipients, while littermate WT mice served as donors for the white adipose tissue. *Procedure*: Both donor and recipient mice were anaesthetised through an intraperitoneal injection of 1% sodium pentobarbital, administered at a dose of 7 µL per gram of body weight. Two pieces of inguinal subcutaneous fat and two pieces of epididymal visceral fat were removed from a single donor mouse via laparotomy. The four fat depots were then divided into eight pieces, each of which was implanted subcutaneously into *Seipin^−/−^* recipient mice using Fuaila medical adhesive. A separate pair of *Seipin^−/−^* and WT control mice underwent the same surgical procedure, but without the AT. Following surgery, mice were housed individually for 1 week and subsequently grouped at 3–4 mice per cage. Twelve weeks after the AT surgery, the mice underwent PHx.

### 2.4 Construction and injection of adeno‐associated virus 8

The adeno‐associated virus 8 (AAV8) system was utilised to induce Seipin overexpress in the mouse liver. Recombinant AAV8 vectors were constructed to carry either human Seipin or a control empty vector, both regulated by a TBG promoter (Serpin A7, a liver‐specific protein). The vectors included AAV–hSeipin (pAAV–TBG–sfGFP–P2A–BSCL2–3×FLAG–WPRE) and AAV–Ctrl (pAAV–TBG–sfGFP–3×FLAG–WPRE), which were produced by Obio Technology Co. Ltd. (Shanghai, China). Eleven‐ to thirteen‐week‐old male *Seipin^−/−^* and littermate WT mice were administered 150 µL of the viral solution via the tail vein, containing 2 × 10^11^ AAV8 vector genomes. Seven days after AAV injection, the mice underwent PHx.

### 2.5 Statistical analysis

Data are presented as mean ± SD. Statistical analyses were performed using GraphPad Prism 9.0 software. For comparisons between two groups, a two‐tailed Student's *t*‐test was employed. One‐way ANOVA followed by Tukey's multiple comparison test was used for comparisons involving more than two groups. For comparisons with multiple variables, two‐way ANOVA was conducted, followed by Sidak's or Tukey's multiple comparison test as appropriate. A *p* value of <.05 was considered statistically significant.

Additional information on key material and methods can be found in the Supporting Information.

## 3 RESULTS

### 3.1 Impaired TRAS and reduced LRR in lipodystrophic *Seipin^−/−^* mice post‐PHx

**FIGURE 1:**
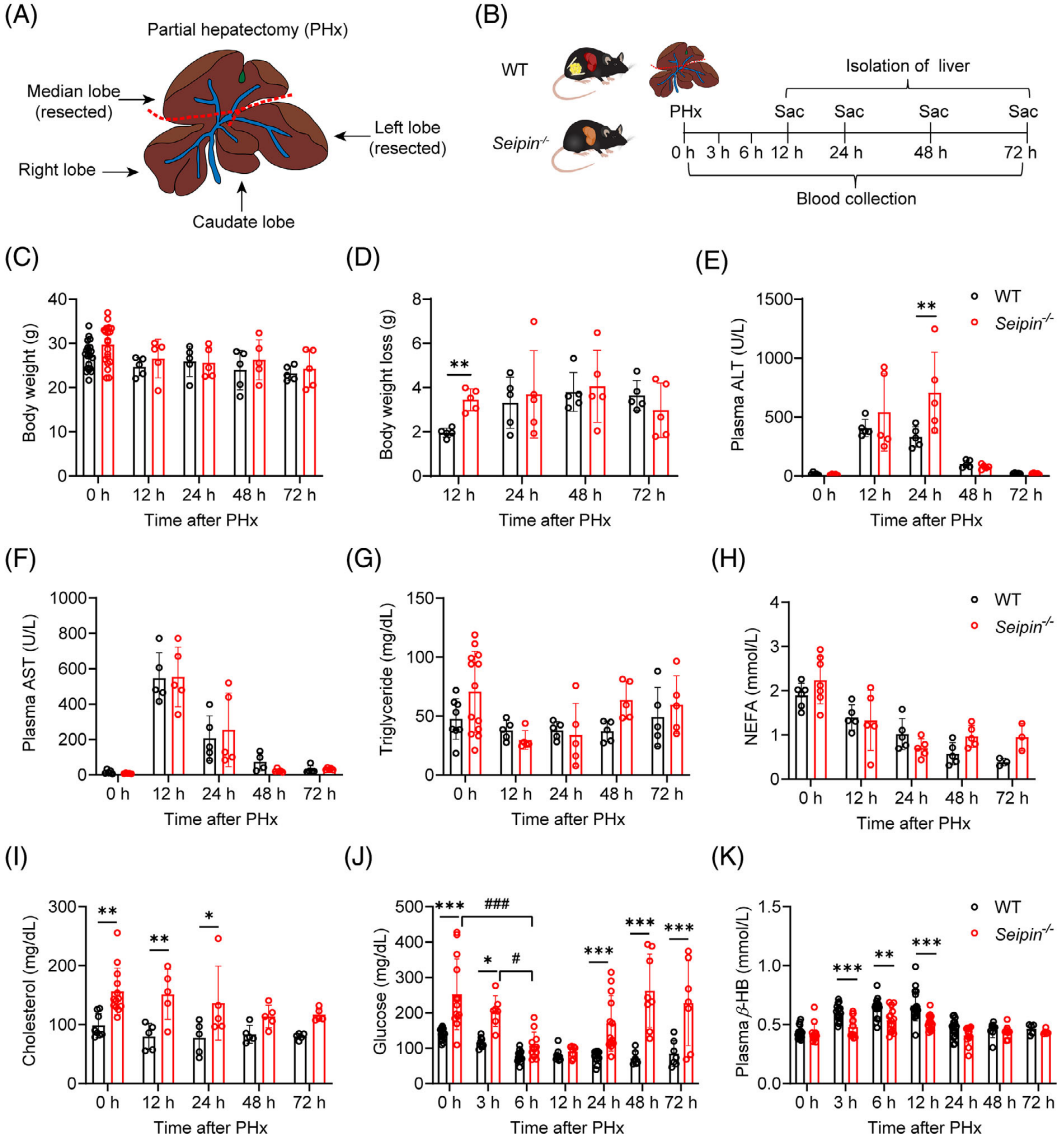
Schematic illustration of partial hepatectomy (PHx) and physiological and biochemical analysis of WT and *Seipin^−/−^* mice post‐PHx. (A) Schematic representation of partial hepatectomy (PHx). A 2/3 partial hepatectomy was performed, with the median and left liver lobes of the mouse removed as indicated by the red dashed line. (B) Experimental timeline. WT and *Seipin^−/−^* mice were subjected to 2/3 PHx. Blood samples were collected at 0, 3, 6, 12, 24, 48 and 72 h after PHx, and liver tissue was isolated at 0, 12, 24, 48 and 72 h post‐PHx. Mice were in the fed state. (C) Body weight of WT and *Seipin^−/−^* mice at 0, 12, 24, 48 and 72 h post‐PHx (*n* = 5–20). Data are shown as mean ± SD. Statistical significance was determined using two‐way ANOVA with Sidak's multiple comparison test. (D) Body weight loss of WT and *Seipin^−/−^* mice at 12, 24, 48 and 72 h after PHx, calculated as the reduction in body weight compared with pre‐PHx levels (*n* = 5). Data are presented as mean ± SD. Statistical significance was determined using two‐way ANOVA with Sidak's multiple comparison test. **p < .01. (E and F) Plasma ALT (E) and AST (F) levels in WT and *Seipin^−/−^* mice at 0, 12, 24, 48 and 72 h after PHx (*n* = 5). Data are shown as mean ± SD. Statistical significance was determined by two‐way ANOVA with Sidak's multiple comparison test. **p < .01. (G–I) Plasma triglycerides (G) (*n* = 5–13), non‐esterified fatty acids (NEFA) (H) (*n* = 5–7) and total cholesterol (I) (*n* = 5–13) levels in WT and *Seipin^−/−^* mice at 0, 12, 24, 48 and 72 h after PHx, measured using enzymatic kits. Data are presented as mean ± SD. Statistical significance was determined by two‐way ANOVA with Sidak's multiple comparison test. *p < .05, **p < .01. (J and K) Plasma glucose (J) (*n* = 7–13) and β‐hydroxybutyrate (K) (*n* = 5–17) levels in WT and *Seipin^−/−^* mice at 0, 3, 6, 12, 24, 48 and 72 h after PHx, measured using enzymatic kits. Data are presented as mean ± SD. Statistical significance was determined by two‐way ANOVA with Sidak's multiple comparison test. *p < .05, **p < .01, ***p < .001 (WT vs. *Seipin^−/−^*), ^#^ *p* < .05 (*Seipin^−/−^*: 3 vs. 6 h), ^###^ *p *< .001 (*Seipin^−/−^*: 0 h vs. 6 h).

To investigate the role of adipose tissue in TRAS and liver regeneration, we employed Seipin knockout mice (*Seipin^−/−^*), which lack adipose tissue. A 2/3 PHx was performed, and the outcomes were compared with WT littermate controls. Plasma samples were collected at 0, 3, 6, 12, 24, 48 and 72 h post‐surgery, and livers were isolated at 0, 12, 24, 48 and 72 h after surgery (Figure 1A,B). No significant differences in body weight, plasma AST, triglyceride or non‐esterified fatty acid (NEFA) levels were observed between WT and *Seipin^−/−^* mice post‐PHx (Figure 1C,F–H). However, *Seipin^−/−^* mice experienced a greater body weight loss compared with WT mice at 12 h post‐PHx (Figure 1D). Plasma ALT levels were higher in *Seipin^−/−^* mice at 24 h post‐PHx, suggesting more severe liver damage compared with WT mice (Figure 1E). Prior to PHx and at 12 and 24 h post‐PHx, *Seipin^−/−^* mice exhibited mild hypercholesterolemia (Figure 1I). Studies have shown that PHx induces early hypoglycaemia, which triggers the release of fatty acids from peripheral lipid reserves, contributing to TRAS during liver regeneration.^12^, ^29^, ^30^ In *Seipin^−/−^* mice, although plasma glucose levels were higher than WT mice at 3 h post‐PHx, they were lower than at baseline (0 h). By 6 h post‐PHx, plasma glucose levels were comparable between *Seipin^−/−^* and WT mice, with both groups showing lower than at 3 h post‐PHx. These results suggest that *Seipin^−/−^* mice, like WT mice, transition to hypoglycaemia after PHx (Figure 1J). Additionally, plasma β‐hydroxybutyrate (β‐HB) levels were significantly lower in *Seipin^−/−^* mice compared with WT mice at 3, 6 and 12 h post‐PHx (Figure 1K).

**FIGURE 2:**
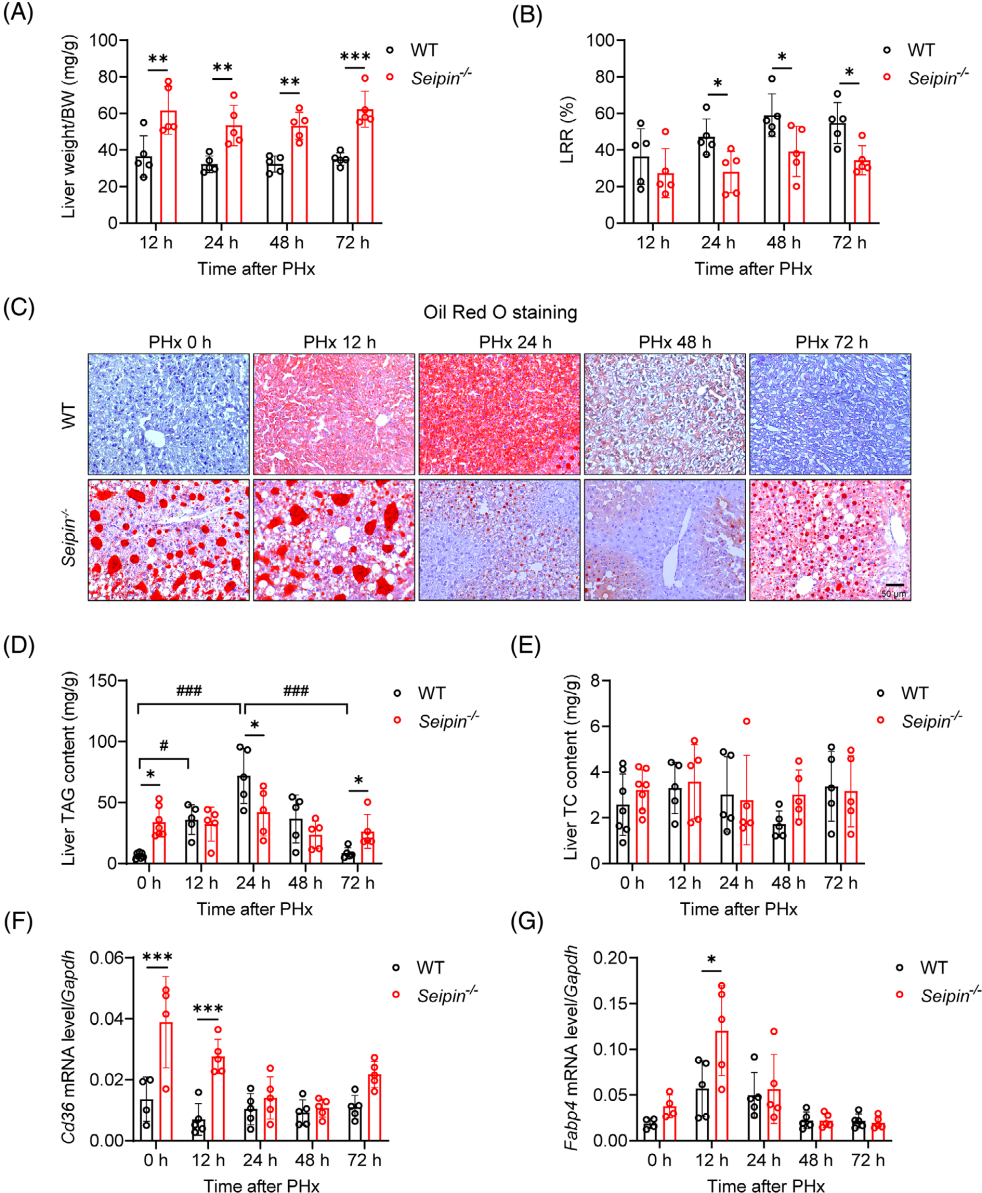
*Seipin^−/−^* mice show impaired transient lipid deposition and reduced liver regeneration after PHx. (A) Ratio of liver weight to body weight in WT and *Seipin^−/−^* mice at 12, 24, 48 and 72 h post‐PHx (*n* = 5). Data are presented as mean ± SD. Statistical significance was determined by two‐way ANOVA with Sidak's multiple comparison test. **p < .01, ***p < .001. (B) Liver regeneration rate (LRR) in WT and *Seipin^−/−^* mice at 12, 24, 48 and 72 h after PHx (*n* = 5). Data are shown as mean ± SD. Statistical significance was determined by two‐way ANOVA with Tukey's multiple comparison test. *p < .05. (C) Representative Oil Red O staining images of liver sections from WT and *Seipin^−/−^* mice at 0, 12, 24, 48 and 72 h after PHx. Scale bar = 50 µm. (D and E) Liver triglyceride (D) and total cholesterol (E) levels in WT and *Seipin^−/−^* mice at 0, 12, 24, 48 and 72 h after PHx, measured using enzymatic kits (*n* = 5–7). Data are shown as mean ± SD. Statistical significance was determined by two‐way ANOVA with Tukey's multiple comparison test. *p < .05 (WT vs. *Seipin^−/−^*), ^#^ *p* < .05 (WT: 0 vs. 12 h), ^###^ *p *< .001 (WT: 0 vs. 24 h, 24 vs. 72 h). (F and G) Relative mRNA expression levels of *Cd36* (F) and *Fabp4* (G) in the liver of WT and *Seipin^−/‐^* mice at 0, 12, 24, 48 and 72 h post‐PHx (*n* = 4–5). Data are presented as mean ± SD. Statistical significance was determined by two‐way ANOVA with Sidak's multiple comparison test. *p < .05, ***p < .001.

To assess liver regeneration, liver weights and regeneration rates were measured. Although *Seipin^−/−^* mice had significantly higher liver weight‐to‐body weight ratios at 12–72 h post‐PHx due to systemic adipose tissue deficiency, the LRRs at 24, 48 and 72 h post‐PHx were significantly lower in *Seipin^−/−^* mice compared with WT mice, indicating impaired liver regeneration (Figure 2A,B). In the early stages of liver regeneration, hepatocytes transiently accumulate large amounts of triglycerides (TRAS) to provide the energy needed for subsequent cell proliferation. To investigate transient lipid accumulation, liver tissues were analysed by Oil Red O staining and lipid content measurements. WT mice showed significant lipid accumulation at 12 h post‐PHx, peaking at 24 h, partially recovering at 48 h, and nearly fully recovering by 72 h, which is the characteristic of TRAS (Figure 2C). These findings were consistent with changes in liver triglyceride content (Figure 2D). In contrast, *Seipin^−/−^* mice exhibited severe liver lipid accumulation both before and at 12 h post‐PHx. However, by 24 and 48 h post‐PHx, there was a reduction in the number of large lipid droplets in *Seipin^−/−^* mice, with levels approaching pre‐surgery conditions by 72 h (Figure 2C). Despite these changes, liver triglyceride content in *Seipin^−/−^* mice did not show significant differences at different time points post‐PHx (Figure 2D). Total cholesterol levels in the liver did not differ significantly between *Seipin^−/−^* and WT mice (Figure 2E). We further analysed mRNA expression of genes related to lipid metabolism: fatty acid uptake (*Cd36*, *Fabp4*), synthesis (*Scd1*, *Acc1*), fatty acid oxidation (*Acox1*) and lipolysis (*Atgl*). *Cd36* and *Fabp4* mRNA levels were significantly higher in *Seipin^−/−^* mice compared with WT mice both before and at 12 h post‐PHx. However, these levels did not differ significantly between the two groups at 24 to 72 h post‐PHx (Figure 2F,G). *Scd1* mRNA levels, indicative of lipid synthesis, were consistently higher in *Seipin^−/−^* mice throughout the time points examined. Although *Acox1* and *Atgl* mRNA levels were lower in *Seipin^−/−^* mice prior to PHx, no significant differences were observed post‐PHx (Figure S1A–E).

In summary, these results demonstrate that *Seipin^−/−^* mice exhibit impaired TRAS and reduced liver regeneration following PHx.

### 3.2 Delayed hepatocyte proliferation in lipodystrophic *Seipin^−/−^* mice post‐PHx

**FIGURE 3:**
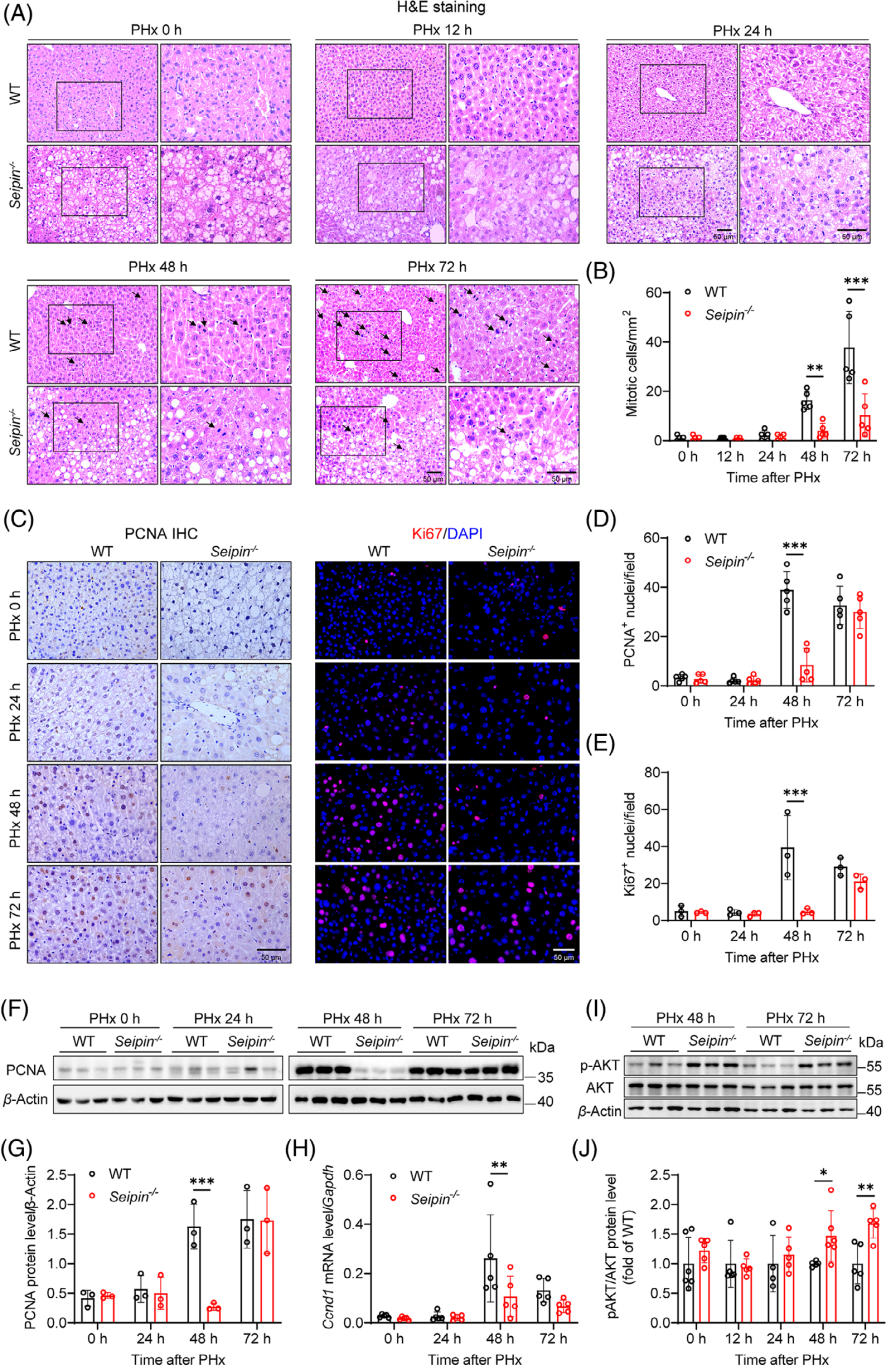
*Seipin^−/−^* mice show delayed hepatocyte proliferation after PHx. (A) Representative H&E staining images showing mitotic cells in liver sections from WT and *Seipin^−/−^* mice at 0, 12, 24, 48 and 72 h after PHx. Images are shown at 200× (left) and 400× (right) magnification. Black arrows indicate mitotic figures. Scale bar = 50 µm. (B) Quantification of mitotic cells from panel A, showing the number of mitotic cells per square millimetre (/mm^2^) in liver sections from WT and *Seipin^−/−^* mice at 0, 12, 24, 48 and 72 h after PHx (*n* = 5). Data are presented as mean ± SD. Statistical significance was determined by two‐way ANOVA with Sidak's multiple comparison test. **p < .01, ***p < .001. (C) Representative immunohistochemical or immunofluorescence images showing PCNA or Ki67‐positive nuclei in liver sections from WT and *Seipin^−/−^* mice at 0, 24, 48 and 72 h after PHx. Scale bar = 50 µm. (D and E) Quantification of PCNA (D) or Ki67 (E) positive nuclei from panel C, showing the number of PCNA or Ki67‐positive nuclei in liver sections from WT and *Seipin^−/−^* mice at 0, 24, 48 and 72 h after PHx (*n* = 3–5). Data are presented as mean ± SD. Statistical significance was determined by two‐way ANOVA with Sidak's multiple comparison test. ***p < .001. (F) Western blot analysis of PCNA protein levels in the liver of WT and *Seipin^−/−^* mice at 0, 24, 48 and 72 h after PHx (*n* = 3). (G) Quantitative analysis of PCNA protein expression levels in the liver of WT and *Seipin^−/−^* mice at 0, 24, 48 and 72 h after PHx (*n* = 3). Data are shown as mean ± SD. Statistical significance was determined by two‐way ANOVA with Sidak's multiple comparison test. ***p < .001. (H) Relative mRNA expression levels of *Ccnd1* in the liver of WT and *Seipin^−/‐^* mice at 0, 24, 48 and 72 h post‐PHx (*n* = 5). Data are presented as mean ± SD. Statistical significance was determined by two‐way ANOVA with Sidak's multiple comparison test. **p < .01. (I) Western blot analysis of pAKT and AKT protein levels in the liver of WT and *Seipin^−/−^* mice at 48 and 72 h post‐PHx. (J) Quantitative analysis of the pAKT/AKT ratio protein expression levels in the liver of WT and *Seipin^/^* mice at 0, 12, 24, 48 and 72 h post‐PHx (*n* = 4–6). Statistical significance was determined by two‐tailed Student's *t*‐test. *p < .05, **p < .01.

To evaluate the effect of Seipin deficiency on hepatocyte proliferation following PHx, we performed H&E staining and pathological analysis of liver tissues collected before and at 12, 24, 48 and 72 h post‐surgery (Figure 3A). Prior to PHx, WT mice exhibited normal liver lobular architecture, while *Seipin^−/−^* mice showed noticeable steatosis. At 24 h post‐PHx, WT liver tissues displayed significant fatty degeneration; in contrast, *Seipin^−/−^* mice had fewer signs of steatosis compared with pre‐surgery levels. By 48 and 72 h post‐PHx, WT mice demonstrated a marked increase in mitotic cells, whereas *Seipin^−/−^* mice had significantly fewer mitotic cells compared with WT mice (Figure 3A,B). Additionally, immunohistochemical staining for proliferating cell nuclear antigen (PCNA), a marker of hepatocyte proliferation, was performed on liver tissues collected at pre‐PHx and at 24, 48 and 72 h post‐PHx (Figure 3C,D). The results revealed a substantial increase in PCNA‐positive proliferating cells in WT mice at 48 and 72 h post‐PHx. Conversely, *Seipin^−/−^* mice had significantly fewer PCNA‐positive cells compared with WT mice at 48 h post‐PHx, with levels recovering to similar values as WT mice by 72 h (Figure 3C,D). Western blot analysis of PCNA protein levels confirmed these findings (Figure 3F,G). Ki67, another marker of hepatocyte proliferation, was detected by immunofluorescence staining, and the results showed a similar trend to the PCNA staining (Figure 3C,E). We also examined the mRNA expression of the cell cycle–related gene *Ccnd1* to further characterise the delayed DNA replication in *Seipin^−/−^* mice. *Ccnd1* was significantly induced 48 h after PHx in WT mice, whereas *Seipin^−/−^* mice exhibited significantly reduced *Ccnd1* mRNA levels at this time point compared with WT. By 72 h post‐PHx, *Ccnd1* mRNA levels were comparable between the two groups (Figure 3H). These observations collectively suggest that hepatocyte proliferation and liver regeneration are delayed in *Seipin^−/−^* mice following PHx.

*Seipin^−/−^* mice also displayed noticeable insulin resistance^22^, ^26^ with significantly lower insulin signalling‐related gene expression (*Irs1* and *Akt2*) in the liver compared with WT mice prior to PHx (Figure S1F). However, 12–48 h after PHx, there were no significant differences in the expression of insulin signalling‐related genes between *Seipin^−/−^* and WT mice (Figure S1G–I). Notably, at 48 and 72 h post‐PHx, the levels of the key insulin signalling protein pAKT/AKT in the liver of *Seipin^−/−^* mice were significantly higher than those in WT mice, indicating enhanced insulin signalling (Figures 3I,J and S1K). These findings suggest that the delay in hepatocyte proliferation observed in *Seipin^−/−^* mice may not be related to insulin signalling, although we cannot rule out the potential negative effect of pre‐existing insulin resistance in *Seipin^−/−^* mice.

### 3.3 AT rescues impaired TRAS and LRR in *Seipin^−/−^* mice

**FIGURE 4:**
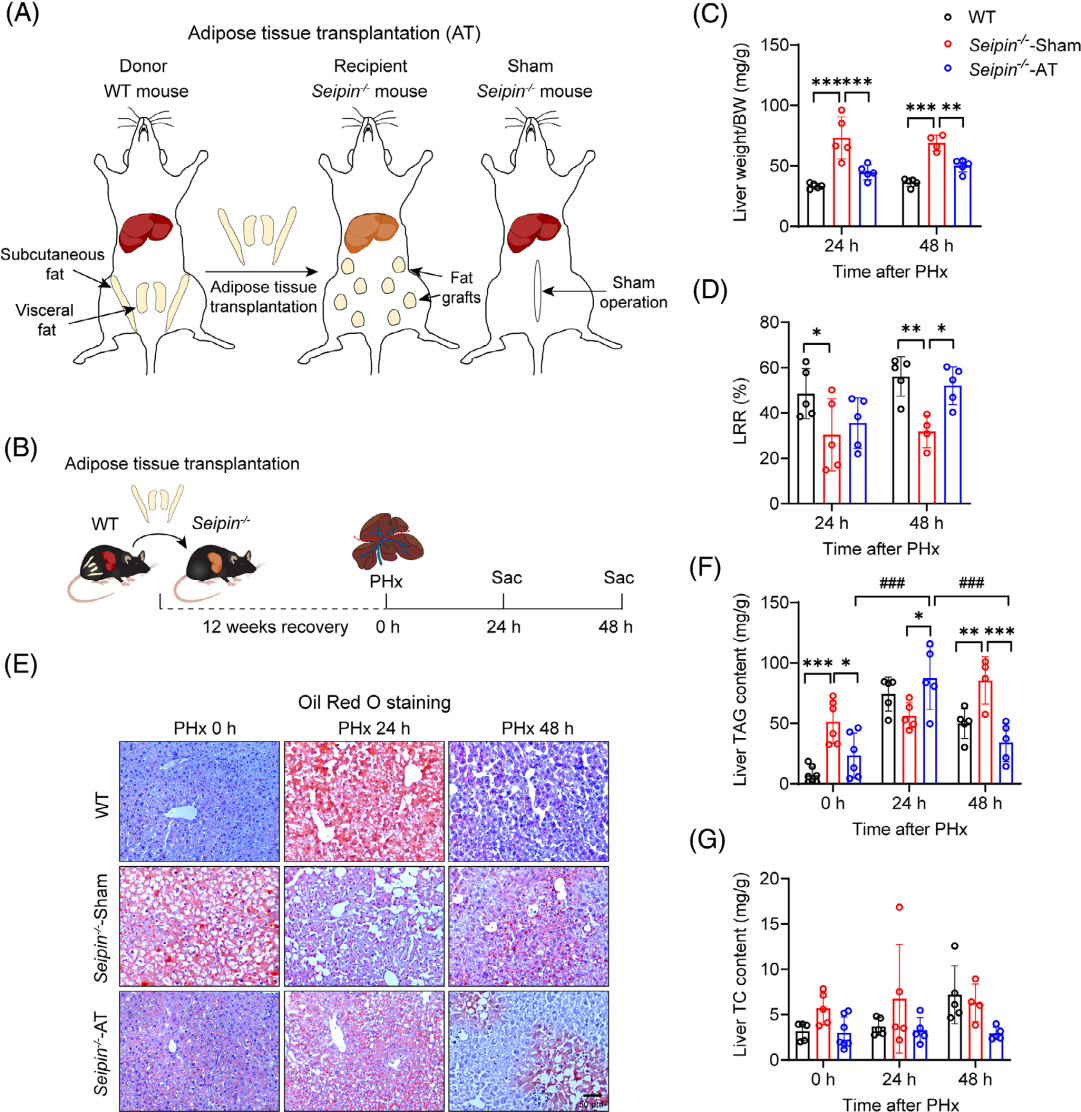
Adipose tissue transplantation rescues impaired TRAS and liver regeneration rate in *Seipin^−/−^* mice. (A) Schematic diagram of the adipose tissue transplantation (AT) procedure. Subcutaneous and visceral fat depots from the inguinal and epididymal regions of WT mice were transplanted subcutaneously into *Seipin^−/−^* mice, generating *Seipin^−/−^*‐AT mice. (B) Experimental timeline. WT, *Seipin^−/−^*‐Sham and *Seipin^−/−^*‐AT underwent AT or Sham operation were recovered for 12 weeks, then subjected to PHx. Blood and liver tissue were collected at 0, 24 and 48 h after PHx. Mice were in the fed state. (C) Ratio of liver weight to body weight in WT, *Seipin^−/−^*‐Sham and *Seipin^−/−^*‐AT mice at 24 and 48 h after PHx (*n* = 4–5). Data are presented as mean ± SD. Statistical significance was determined by two‐way ANOVA with Sidak's multiple comparison test. **p < .01, ***p < .001. (D) Liver regeneration rate (LRR) in WT, *Seipin^−/−^*‐Sham and *Seipin^−/−^*‐AT mice at 24 and 48 h after PHx (*n* = 4–5). Data are shown as mean ± SD. Statistical significance was determined by two‐way ANOVA with Tukey's multiple comparison test. *p < .05, **p < .01. (E) Representative Oil Red O staining images of liver sections from WT, *Seipin^−/−^*‐Sham and *Seipin^−/−^*‐AT mice at 0, 24 and 48 h after PHx. Scale bar = 50 µm. (F and G) Liver triglyceride (F) and total cholesterol (G) levels in WT, *Seipin^−/−^*‐Sham and *Seipin^−/−^*‐AT mice at 0, 24 and 48 h after PHx, measured using enzymatic kits (*n* = 4–5). Data are shown as mean ± SD. Statistical significance was determined by two‐way ANOVA with Tukey's multiple comparison test. *p < .05, **p < .01, ***p < .001 (WT vs. *Seipin^−/−^*‐Sham, *Seipin^−/−^*‐Sham vs. *Seipin^−/−^*‐AT), ^###^ *p *< .001 (*Seipin^−/−^*‐AT: 0 vs. 24 h, 24 vs. 48 h).

To determine if the impaired liver regeneration in *Seipin^−/−^* mice is due to the absence of adipose tissue, we performed AT into *Seipin^−/−^* mice and assessed its effects on transient lipid accumulation and liver regeneration after PHx. White adipose tissue from the epididymal and inguinal regions of WT mice was transplanted subcutaneously into *Seipin^−/−^* mice, generating *Seipin^−/−^*‐AT mice (Figure 4A). PHx was performed 12 weeks after AT, and liver and plasma samples were collected 0, 24 and 48 h post‐PHx (Figure 4B). Pre‐PHx liver morphology indicated that fatty liver was significantly improved in *Seipin^−/−^*‐AT mice compared with *Seipin^−/−^*‐Sham mice (Figure S2A). The viability of the transplanted adipose tissue was confirmed in *Seipin^−/−^*‐AT mice at the time of sampling (Figure S2B). Body weight, body weight loss, plasma ALT, AST, triglycerides, cholesterol and glucose levels were measured pre‐PHx and at 24 and 48 h post‐PHx (Figure S2C–I). ALT levels were lower in *Seipin^−/−^*‐AT mice at 24 h post‐PHx compared with *Seipin^−/−^*‐Sham mice (Figure S2E), and plasma cholesterol and glucose levels were also significantly improved (Figure S2H,I).

Liver weight and regeneration rates were assessed to evaluate the impact of AT on liver regeneration in *Seipin^−/−^* mice. Liver weight in *Seipin^−/−^*‐AT mice was significantly lower than in *Seipin^−/−^*‐Sham mice, consistent with the observed improvement in fatty liver (Figure 4C). Remarkably, LRRs were significantly higher in *Seipin^−/−^*‐AT mice at 48 h post‐PHx compared with *Seipin^−/−^*‐Sham mice (Figure 4D). To evaluate the effect of AT on TRAS after PHx, liver tissues were subjected to Oil Red O staining, and liver triglyceride and cholesterol levels were measured (Figure 4E–G). Pre‐PHx liver tissue from *Seipin^−/−^*‐AT mice showed significantly less Oil Red O‐positive lipid staining and lower liver triglyceride levels compared with *Seipin^−/−^*‐Sham mice, indicating substantial improvement in fatty liver (Figure 4E,F). *Seipin^−/−^*‐AT mice exhibited significant lipid accumulation like WT mice at 24 h post‐PHx, with liver triglyceride levels significantly higher than pre‐PHx levels. By 48 h post‐PHx, lipid accumulation in *Seipin^−/−^*‐AT mice had partially recovered (Figure 4E,F). These findings demonstrate that AT effectively corrects impaired TRAS and enhances liver regeneration in *Seipin^−/−^* mice following PHx.

### 3.4 AT rescues delayed hepatocyte proliferation in *Seipin^−/−^* mice

**FIGURE 5:**
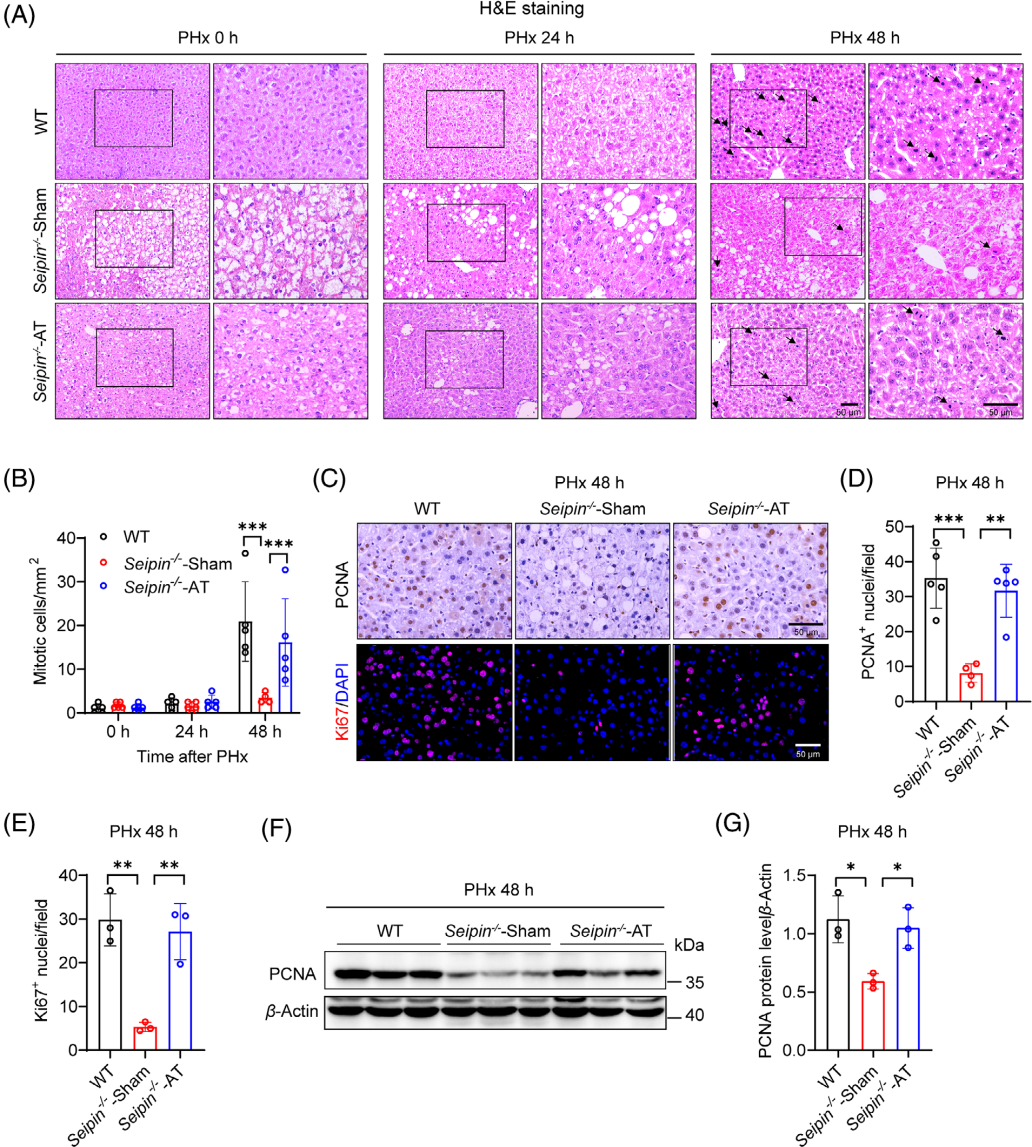
Adipose tissue transplantation rescues delayed hepatocyte proliferation in *Seipin^−/−^* Mice. (A) Representative images of H&E staining illustrating mitotic cells in liver sections from WT, *Seipin^−/−^*‐Sham and *Seipin^−/−^*‐AT 0, 24 and 48 h post‐PHx. Images are shown at 200× (left) and 400× (right) magnification. Black arrows indicate mitotic figures. Scale bar = 50 µm. (B) Quantitative analysis of mitotic cells from panel A. Data represent the number of mitotic cells per square millimetre (/mm^2^) in liver sections from WT, *Seipin^−/−^*‐Sham and *Seipin^−/−^*‐AT mice at 0, 24 and 48 h post‐PHx (*n* = 4–5). Data are presented as mean ± SD. Statistical significance was determined using two‐way ANOVA with Sidak's multiple comparison test. ***p < .001. (C) Representative images of immunohistochemical or immunofluorescence staining for PCNA or Ki67‐positive nuclei in liver sections from WT, *Seipin^−/−^*‐Sham and *Seipin^−/−^*‐AT mice at 48 h post‐PHx. Scale bar = 50 µm. (D and E) Quantitative analysis of PCNA (D) or Ki67 (E) positive nuclei from panel C. The data represent the number of PCNA or Ki67‐positive nuclei per field at 400× magnification in liver sections from WT, *Seipin^−/−^*‐Sham and *Seipin^−/−^*‐AT mice at 48 h post‐PHx (*n* = 3–5). Data are presented as mean ± SD. Statistical significance was determined using one‐way ANOVA with Tukey's multiple comparison test. **p < .01, ***p < .001. (F) Western blot analysis of PCNA protein levels in the liver of WT, *Seipin^−/−^*‐Sham and *Seipin^−/−^*‐AT mice at 48 h post‐PHx (*n* = 3). (G) Quantitative analysis of PCNA protein expression levels in the liver of WT, *Seipin^−/−^*‐Sham and *Seipin^−/−^*‐AT mice at 48 h post‐PHx (*n* = 3). Data are presented as mean ± SD. Statistical significance was determined using one‐way ANOVA with Tukey's multiple comparison test. *p < .05.

To assess the impact of AT on hepatocyte proliferation in *Seipin^−/−^* mice, we analysed liver tissues from WT, *Seipin^−/−^*‐Sham and *Seipin^−/−^*‐AT mice before and 24 and 48 h after PHx using H&E staining and mitotic cell counts. Prior to PHx, *Seipin^−/−^*‐Sham mice liver exhibited significant steatosis, whereas *Seipin^−/−^*‐AT mice showed notable improvement (Figure 5A). At 48 h post‐PHx, WT mice displayed a substantial increase in mitotic cells, while *Seipin^−/−^*‐Sham mice had fewer mitotic cells. In contrast, *Seipin^−/−^*‐AT mice showed a significant increase in mitotic cells compared with *Seipin^−/−^*‐Sham mice (Figure 5A,B). Additionally, liver tissues from all three groups at 48 h post‐PHx were subjected to PCNA immunohistochemical, Ki67 immunofluorescence staining and PCNA Western blot analysis to assess hepatocyte proliferation. The results revealed that PCNA and Ki67 levels were significantly lower in *Seipin^−/−^*‐Sham mice compared with WT mice at 48 h post‐PHx. In contrast, PCNA and Ki67 levels in *Seipin^−/−^*‐AT mice were significantly higher than in *Seipin^−/−^*‐Sham mice, approaching those of WT mice (Figure 5C–G). These findings indicate that AT effectively restores the delayed hepatocyte proliferation observed in *Seipin^−/−^* mice.

### 3.5 Liver‐specific human Seipin overexpression does not affect TRAS and liver regeneration post‐PHx

**FIGURE 6:**
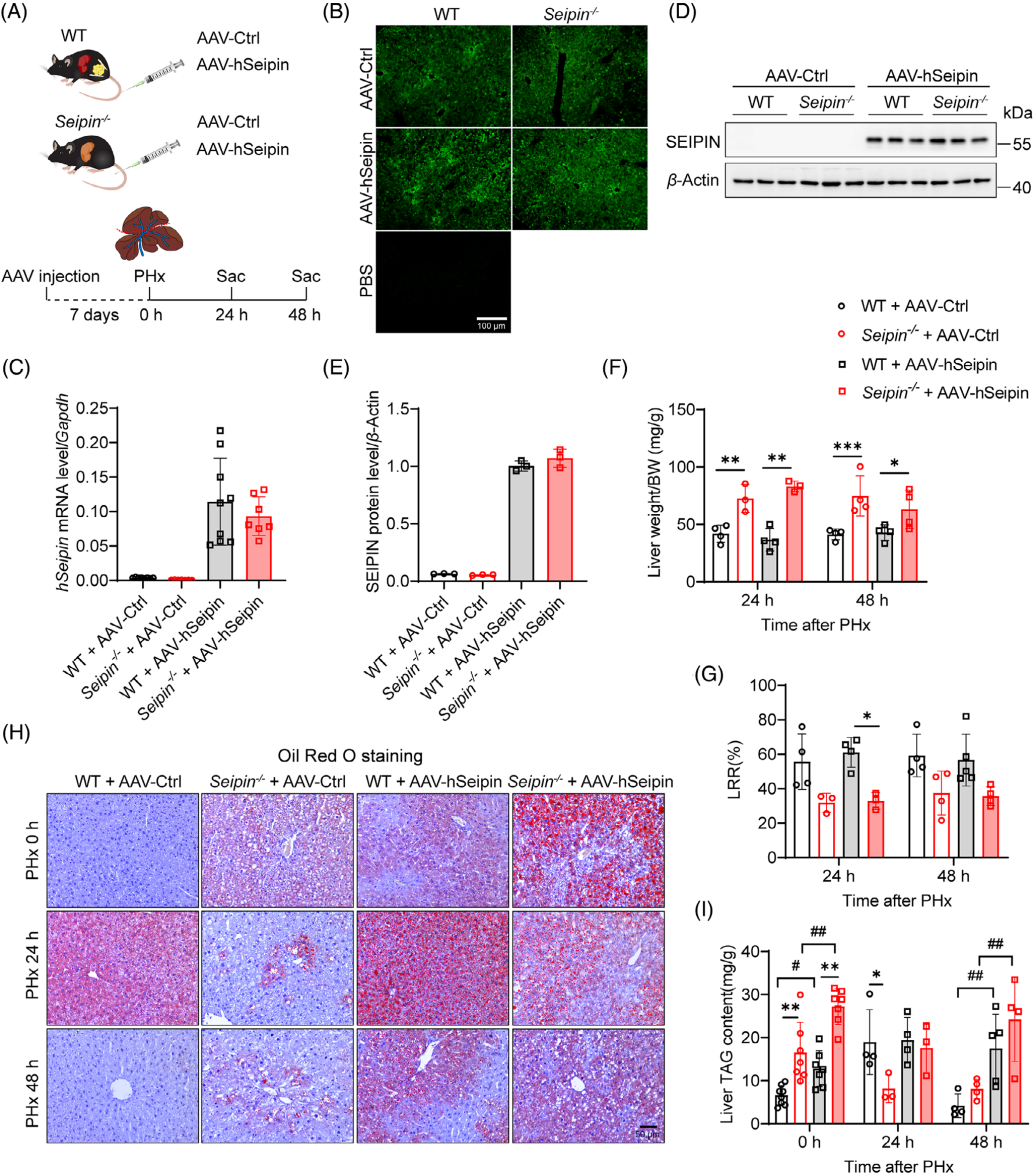
Liver‐specific overexpression of Seipin does not affect TRAS and liver regeneration rate in both WT and *Seipin^−/−^* Mice post‐PHx. (A) Experimental timeline for liver‐specific overexpression of Seipin in WT and *Seipin^−/−^* mice. Mice were injected with 2 × 10^11^ vg AAV8–TBG–GFP (AAV–Ctrl) or AAV8–TBG–hSeipin (AAV–hSeipin) via tail vein. Partial hepatectomy (PHx) was performed 7 days after AAV injection. Blood and liver tissue were collected at 0, 24 and 48 h after PHx. Mice were in the fed state. (B) Representative images of green fluorescent protein (GFP) observed via fluorescence microscopy in liver tissue from WT + AAV–Ctrl, *Seipin^−/−^* + AAV–Ctrl, WT + AAV–hSeipin and *Seipin^−/−^* + AAV–hSeipin mice. Scale bar = 100 µm. (C) RT‐qPCR analysis of relative *hSeipin* mRNA expression in the liver of WT + AAV–Ctrl, *Seipin^−/−^* + AAV–Ctrl, WT + AAV–hSeipin and *Seipin^−/−^* + AAV–hSeipin mice (*n* = 7–9). (D) Western blot analysis of Seipin protein levels in the liver of WT + AAV–Ctrl, *Seipin^−/−^* + AAV–Ctrl, WT + AAV–hSeipin and *Seipin^−/−^* + AAV–hSeipin mice 7 days post‐AAV infection (*n* = 3). (E) Quantitative analysis of Seipin protein expression levels in the liver of WT + AAV–Ctrl, *Seipin^−/−^* + AAV–Ctrl, WT + AAV–hSeipin and *Seipin^−/−^* + AAV–hSeipin mice (*n* = 3). (F) Ratio of liver weight to body weight in WT + AAV–Ctrl, *Seipin^−/−^* + AAV–Ctrl, WT + AAV–hSeipin and *Seipin^−/−^* + AAV–hSeipin mice at 24 and 48 h post‐PHx (*n* = 3–4). Data are presented as mean ± SD. Statistical significance was determined using two‐way ANOVA with Sidak's multiple comparison test. *p < .05, **p < .01, ***p < .001. (G) Liver regeneration rate (LRR) in WT + AAV–Ctrl, *Seipin^−/−^* + AAV–Ctrl, WT + AAV–hSeipin and *Seipin^−/−^* + AAV–hSeipin mice at 24 and 48 h post‐PHx (*n* = 3–5). Data are presented as mean ± SD. Statistical significance was determined using two‐way ANOVA with Sidak's multiple comparison test. *p < .05. (H) Representative images of Oil Red O staining in liver sections from WT + AAV–Ctrl, *Seipin^−/−^* + AAV–Ctrl, WT + AAV–hSeipin and *Seipin^−/−^* + AAV–hSeipin mice at 0, 24 and 48 h post‐PHx. Scale bar = 50 µm. (I) Liver triglyceride levels in WT + AAV–Ctrl, *Seipin^−/−^* + AAV–Ctrl, WT + AAV–hSeipin and *Seipin^−/−^* + AAV–hSeipin mice at 0, 24 and 48 h post‐PHx, measured using enzymatic kits (*n* = 3–9). Data are presented as mean ± SD. Statistical significance was determined using two‐way ANOVA with Tukey's multiple comparison test. *p < .05, **p < .01 (WT vs. *Seipin^−/−^*), ^#^ *p* < .05, ^##^ *p* < .01 (AAV–Ctrl vs. AAV–hSeipin).

To further determine whether the impaired TRAS and delayed hepatocyte proliferation observed in *Seipin^−/−^* mice after PHx are due to the loss of adipose tissue rather than the effects of Seipin protein itself, we used an AAV8 vector with a hepatocyte‐specific TBG promoter to overexpress human Seipin in the liver of WT and *Seipin^−/−^* mice. PHx was performed 7 days after AAV injection, and liver and plasma samples were collected before PHx, as well as at 24 and 48 h post‐PHx (Figures 6A and S3A). Fluorescence microscopy confirmed successful expression of the AAV8 vectors in the liver, with evident green fluorescence in liver tissues from both AAV–hSeipin and control virus (AAV–Ctrl) injected mice (Figure 6B). Quantitative real‐time PCR and Western blot analyses demonstrated a significant increase in Seipin mRNA and protein levels in liver tissues of WT + AAV–hSeipin and *Seipin^−/−^* + AAV–hSeipin mice (Figure 6C–E).

**FIGURE 7:**
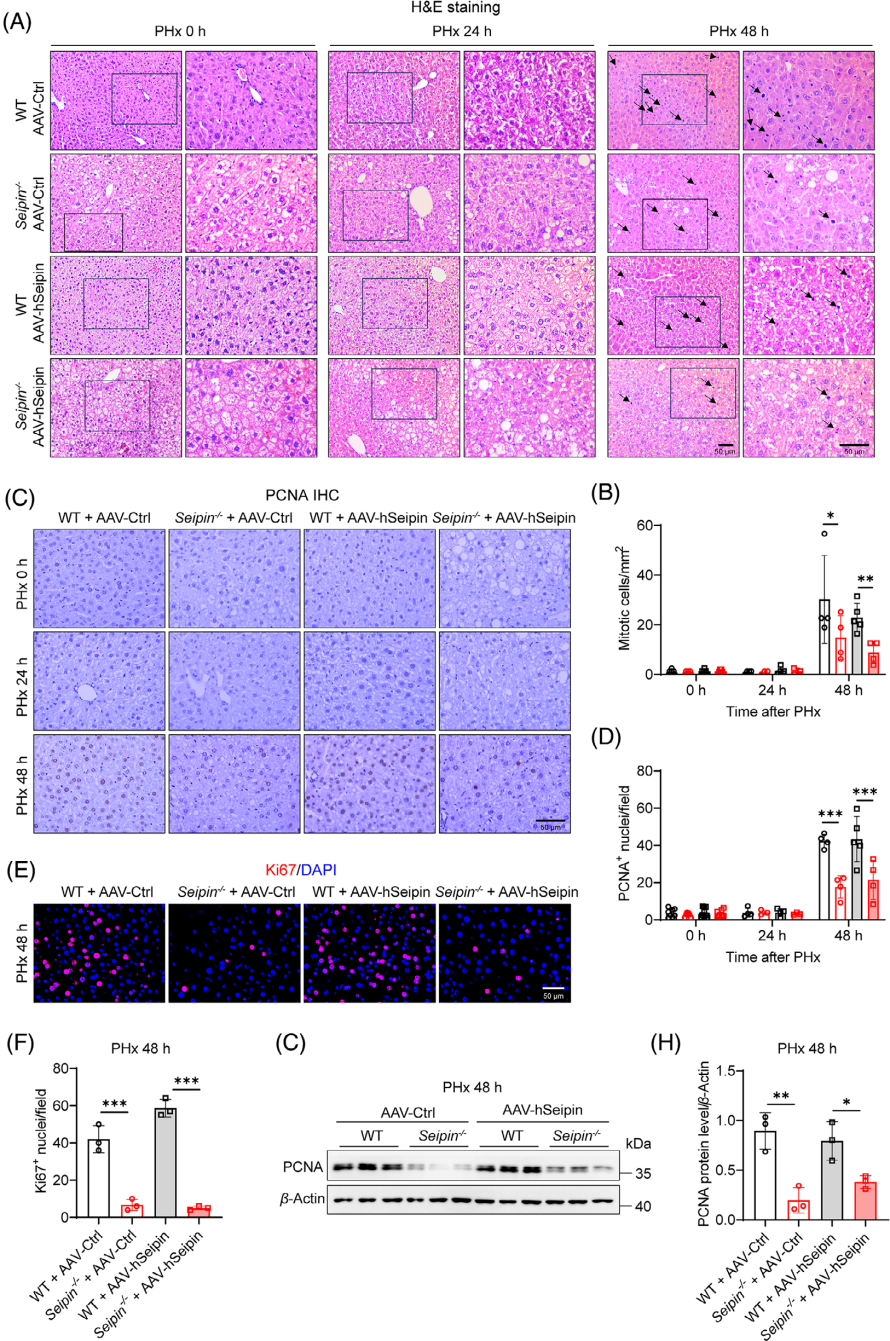
Liver‐specific overexpression of Seipin does not affect hepatocyte proliferation in both WT and *Seipin^−/−^* Mice post‐PHx. (A) Representative images of H&E staining showing mitotic cells in liver sections from WT + AAV–Ctrl, *Seipin^−/−^* + AAV–Ctrl, WT + AAV–hSeipin and *Seipin^−/−^* + AAV–hSeipin mice at 0, 24 and 48 h post‐PHx. Images are shown at 200× (left) and 400× (right) magnification. Black arrows indicate mitotic figures. Scale bar = 50 µm. (B) Quantitative analysis of mitotic cells from panel A. Data show the number of mitotic cells per square millimetre (/mm^2^) in liver sections from WT + AAV–Ctrl, *Seipin^−/−^* + AAV–Ctrl, WT + AAV–hSeipin and *Seipin^−/−^* + AAV–hSeipin mice at 0, 24 and 48 h post‐PHx (*n* = 3–5). Data are presented as mean ± SD. Statistical significance was determined using two‐way ANOVA with Sidak's multiple comparison test. *p < .05, **p < .01. (C) Representative images of immunohistochemical staining for PCNA‐positive nuclei in liver sections from WT + AAV–Ctrl, *Seipin^−/−^* + AAV–Ctrl, WT + AAV–hSeipin and *Seipin^−/−^* + AAV–hSeipin mice at 0, 24 and 48 h post‐PHx. Scale bar = 50 µm. (D) Quantitative analysis of PCNA‐positive nuclei from panel C. Data show the number of PCNA‐positive nuclei per liver section from WT + AAV–Ctrl, *Seipin^−/−^* + AAV–Ctrl, WT + AAV–hSeipin and *Seipin^−/−^* + AAV–hSeipin mice at 0, 24 and 48 h post‐PHx (*n* = 3–5). Data are presented as mean ± SD. Statistical significance was determined using two‐way ANOVA with Sidak's multiple comparison test. ***p < .001. (E) Representative images of immunofluorescence staining for Ki67‐positive nuclei in liver sections from WT + AAV–Ctrl, *Seipin^−/−^* + AAV–Ctrl, WT + AAV–hSeipin and *Seipin^−/−^* + AAV–hSeipin mice at 48 h post‐PHx. Scale bar = 50 µm. (F) Quantitative analysis of Ki67‐positive nuclei from panel E. Data show the number of Ki67‐positive nuclei per liver section from WT + AAV–Ctrl, *Seipin^−/−^* + AAV–Ctrl, WT + AAV–hSeipin and *Seipin^−/−^* + AAV–hSeipin mice at 48 h post‐PHx (*n* = 3). Data are presented as mean ± SD. Statistical significance was determined using two‐way ANOVA with Sidak's multiple comparison test. ***p < .001. (G) Western blot analysis of PCNA protein levels in the liver of WT + AAV–Ctrl, *Seipin^−/−^* + AAV–Ctrl, WT + AAV–hSeipin and *Seipin^−/−^* + AAV–hSeipin mice at 48 h post‐PHx. (H) Quantitative analysis of PCNA protein expression levels in the liver of WT + AAV–Ctrl, *Seipin^−/−^* + AAV–Ctrl, WT + AAV–hSeipin and *Seipin^−/−^* + AAV–hSeipin mice at 48 h post‐PHx (*n* = 3). Data are presented as mean ± SD. Statistical significance was determined using two‐way ANOVA with Sidak's multiple comparison test. *p < .05, **p < .01.

Liver‐specific Seipin overexpression did not affect liver weight or regeneration rates post‐PHx (Figure 6F,G), neither did it influence body weight, body weight loss, plasma ALT, AST or glucose levels before or at 24 and 48 h after PHx (Figure S3B–E,H). However, it resulted in significantly lower plasma triglyceride and cholesterol levels in both WT and *Seipin^−/−^* mice (Figure S3F,G). Oil Red O staining and liver triglyceride content analysis revealed that, while WT + AAV–hSeipin mice had mild lipid deposition in the liver before PHx, they exhibited significantly increased lipid accumulation at 24 h post‐PHx, with partial restoration by 48 h. This pattern is characteristic of TRAS (Figure 6H,I). Quantitative analysis of mitotic cells in liver tissues indicated that liver‐specific Seipin overexpression did not affect the number of mitotic cells post‐PHx in either WT or *Seipin^−/−^* mice (Figure 7A,B). Immunohistochemical staining of PCNA, immunofluorescence staining of Ki67, and Western blot analysis of PCNA also showed no significant differences between AAV–hSeipin and AAV–Ctrl injected mice in both WT and *Seipin^−/−^* genotypes (Figure 7C–H). These results suggest that liver‐specific Seipin overexpression does not influence hepatocyte proliferation after PHx and could not restore impaired hepatocyte proliferation in *Seipin^−/−^* mice post‐PHx.

Overall, these findings indicate that liver‐specific overexpression of Seipin does not affect transient lipid deposition or liver regeneration in either WT or *Seipin^−/−^* mice following PHx. The impaired TRAS and delayed hepatocyte proliferation observed in *Seipin^−/−^* mice after PHx are not due to the absence of hepatic Seipin protein itself.

## 4 DISCUSSION

This study investigated the role of adipose tissue in liver regeneration after 2/3 PHx injury using Seipin knockout mice (*Seipin^−/−^*), which lack systemic adipose tissue, complemented by normal AT and hepatocyte‐specific Seipin overexpression models. Our findings reveal that Seipin deficiency significantly affects TRAS and hepatocyte proliferation, highlighting the critical role of adipose tissue in these processes. Our results also strongly indicate that adipose tissue, but not hepatic Seipin, is required for normal liver regeneration.

While previous studies have emphasised the importance of adipose tissue lipolysis in TRAS, evidence from animal models of adipose tissue deficiency remains limited. For example, *Caveolin‐1* is a key gene involved in CGL4. Research has shown that *Caveolin‐1* knockout mice have reduced survival rates and decreased hepatic triglyceride accumulation following PHx.^31^, ^32^ However, the accumulation of Caveolin‐1 in hepatocyte lipid droplets post‐PHx, along with its role in regulating hepatocyte proliferation, suggests that these phenotypes might be independent of adipose tissue deficiency. Additionally, fatty liver dystrophy (*fld*) mice with Lipin1 deficiency, which causes partial loss of adipose tissue, exhibit reduced hepatic triglyceride content and inhibited hepatocyte proliferation post‐PHx,^33^ suggesting that reduced adipose tissue suppresses liver regeneration. However, Lipin1‐deficient mice still retain some adipose tissue, and Lipin1 is highly expressed in hepatocytes, where it plays a crucial role in triglyceride synthesis and influences fatty acid oxidation through PPARα.^34^, ^35^ Therefore, the tissue‐autonomous functions of Caveolin‐1 and Lipin1 in hepatocytes may directly impact TRAS and liver regeneration.

Here, by using a unique model (i.e. the *Seipin^−/−^* mice), our study provides the most convincing evidence on the role of adipose tissue in liver regeneration. Seipin is unique in this context because Seipin deficiency causes the most severe form of human lipodystrophy and because Seipin is almost completely absent from hepatocytes.^36^ Moreover, hepatocyte‐specific Seipin knockout mice do not exhibit significant metabolic changes, suggesting that Seipin does not have a cell‐autonomous homeostatic effect in hepatocytes.^22^, ^23^ Therefore, *Seipin^−/−^* mice serve as an ideal model for studying the impact of adipose tissue deficiency on liver metabolic diseases. The transplantation of normal adipose tissue into *Seipin^−/−^* mice further confirms the crucial role of normal adipose tissue in correcting TRAS and hepatocyte proliferation delays. Additionally, liver‐specific Seipin overexpression using AAV viruses did not significantly impact TRAS or liver regeneration in either WT or *Seipin^−/−^* mice, reinforcing the notion that adipose tissue, rather than hepatic Seipin protein itself, is critical for these processes.

Following a 2/3 PHx, WT mice demonstrated substantial lipid accumulation in the liver 24 h after surgery, indicative of robust TRAS. In contrast, *Seipin^−/−^* mice exhibited markedly reduced large lipid droplets in hepatocytes, suggesting impaired TRAS. This reduction may due to the *Seipin^−/−^* mice's inability to mobilise fatty acids from adipose tissue to meet the energy demands during the early post‐PHx period. Consequently, hepatocytes hydrolyse triglycerides stored in lipid droplets, leading to fewer large lipid droplets. By 72 h post‐surgery, the liver's energy needs shifted away from reliance on triglyceride hydrolysis. As a result, *Seipin^−/−^* mice, which lack adipose tissue as a triglyceride storage organ, experienced ectopic lipid accumulation in the liver, resulting in a gradual restoration of hepatic lipid deposits.

Additionally, we observed that WT mice showed a high number of mitotic cells and significant expression of the hepatocyte proliferation marker PCNA, Ki67 and cell cycle related gene *Ccnd1* at 48 and 72 h post‐PHx. In contrast, *Seipin^−/−^* mice had reduced PCNA, Ki67 and *Ccnd1* expression at 48 h post‐PHx, with levels comparable to WT mice by 72 h. This suggests that Seipin deficiency leads to a delay in hepatocyte proliferation, likely due to impaired TRAS. In the absence of Seipin, TRAS is obstructed, preventing adequate energy supply for hepatocyte proliferation.

Previous research has demonstrated that PHx leads to a temporary drop in glucose levels, followed by lipid accumulation in hepatocytes.^29^ It is proposed that early hypoglycaemia triggers the release of fatty acids from peripheral lipid reserves, which then accumulate in hepatocytes and contribute to TRAS during liver regeneration.^12^, ^30^ In *Seipin^−/−^* mice, although they show higher plasma glucose levels than WT mice at 3 h post‐PHx, these levels were lower than at 0 h. At 6 h post‐PHx, plasma glucose levels were comparable between *Seipin^−/−^* and WT mice, suggesting that *Seipin^−/−^* mice, like WT mice, experience a transition to hypoglycaemia after PHx.

Beyond the absence of adipose tissue, it is important to note that *Seipin^−/−^* mice also exhibit severe fatty liver and insulin resistance—well‐established features of lipodystrophy. These factors may also influence liver regeneration. The impact of fatty liver on liver regeneration remains controversial in the literature. While some studies suggest that hepatic steatosis, particularly in the context of steatohepatitis, impairs liver regeneration,^37^ others report no significant effects from models like methionine–choline‐deficient diets.^38^, ^39^ Furthermore, mild hepatic steatosis, such as that induced by a Western diet, may even enhance liver regeneration.^40^ However, severe steatosis is generally considered detrimental to liver regeneration, particularly in clinical transplantation settings.^41^, ^42^ While our study demonstrates delayed liver regeneration in *Seipin^−/−^* mice, the pre‐existing fatty liver in these mice may also contribute to this impairment.

Furthermore, adipose tissue deficiency is frequently linked with severe insulin resistance that can impact hepatocyte function and proliferation signalling pathways.^43^, ^44^ Although *Seipin^−/−^* mice exhibit severe insulin resistance under fed or short‐term fasting conditions,^22^, ^26^ our study found no significant differences in key insulin signalling factors, such as pAKT, or in the mRNA levels of key players in the insulin signalling pathway between *Seipin^−/−^* and WT mice 12–24 h post‐PHx. Interestingly, pAKT levels were significantly elevated in *Seipin^−/−^* mice at 48 and 72 h after PHx compared with WT mice, suggesting that the impaired liver regeneration observed in *Seipin^−/−^* mice may not be due to insulin resistance, although we cannot rule out the potential negative effect of pre‐existing insulin resistance in *Seipin^−/−^* mice. Prolonged fasting, which induces lipolysis, typically results in substantial hepatic lipid accumulation in WT mice. However, prior research has shown that *Seipin^−/−^* mice do not exhibit fasting‐induced hepatic lipid accumulation and actually demonstrate enhanced hepatic insulin sensitivity following prolonged fasting,^22^, ^45^ which aligns with our observation of increased insulin signalling in *Seipin^−/−^* mice 48 h post‐PHx.

Adipose tissue and the liver engage in a complex interaction that influences liver regeneration through several mechanisms, including lipolysis, endocrine signalling, mesenchymal stem cells (MSCs), metabolite exchange and immune regulation. During liver regeneration, lipolysis provides FFAs from adipose tissue, which are crucial for hepatocyte energy production, proliferation and metabolic recovery.^12^, ^46^ In *Seipin^−/−^* mice, impaired FFA release disrupts liver regeneration, highlighting the importance of adipose‐derived fatty acids. Adipose tissue also acts as an endocrine organ, releasing adipokines such as leptin, adiponectin and resistin, which regulate liver function and regeneration. Leptin supplementation inhibits PHx‐induced liver regeneration,^47^ while adiponectin knockout impairs liver regeneration, underscoring the role of these adipokines.^48^, ^49^ Additionally, adipose tissue is a reservoir of MSCs, which contribute to liver repair; adipose‐derived MSCs have shown promise in liver regeneration.^50^, ^51^ In lipodystrophy, dysfunctional adipose tissue may reduce MSC availability, impairing liver recovery. Furthermore, adipose tissue releases extracellular vesicles containing microRNAs, lipids and bioactive molecules that influence liver function.^52^, ^53^ It also releases metabolites, such as glycerol and amino acids, which affect liver metabolism.^54^, ^55^ Adipose tissue plays a crucial role in immune modulation, with dysfunctional adipose tissue in conditions like obesity or lipodystrophy releasing pro‐inflammatory cytokines (e.g., TNF‐α, IL‐6), which alter hepatic inflammation, a key factor in liver regeneration.^56^, ^57^ Thus, adipose tissue is vital for regulating liver recovery after injury.

A limitation of our study is the pre‐existing fatty liver and insulin resistance in *Seipin^−/−^* mice. Performing PHx after AT, while retaining the existing fatty liver and insulin resistance, would more effectively isolate and emphasise the specific role of adipose tissue in liver regeneration. This approach would help clarify the contribution of *Seipin^−/−^*‐induced fatty liver and insulin resistance to the observed delays in liver regeneration. Additionally, future studies could benefit from including other adipose tissue‐deficient mouse models, such as adipose‐specific *Seipin* or *Agpat2* knockout mice, to further strengthen our findings.

## 5 CONCLUSION

This study unveils the critical role of adipose tissue in liver regeneration following PHx by employing Seipin knockout mice as a model of systemic adipose tissue deficiency. Our findings reveal that Seipin deficiency impairs TRAS and delays hepatocyte proliferation, leading to compromised liver regeneration. Importantly, the transplantation of normal adipose tissue into *Seipin^−/−^* mice effectively restores TRAS and enhances hepatocyte proliferation, confirming that adipose tissue is essential for these processes.

## AUTHOR CONTRIBUTIONS

*Investigation, methodology, data curation, validation, visualisation and writing – original draft*: Qianqian Dong. *Investigation, methodology, data curation, validation and visualisation*: Ziwei Liu. *Methodology, data curation and visualisation*: Yidan Ma. *Investigation, methodology, data curation, validation and visualisation*: Xin Chen. *Investigation, methodology, visualisation and software*: Xiao Wang. *Investigation and methodology*: Jinye Tang. *Investigation and methodology*: Kexin Ma. *Investigation and methodology*: Chenxi Liang. *Methodology*: Mengyu Wang. *Systematic review and visualisation*: Xiaoqin Wu. *Systematic review and visualisation*: Yang Liu. *Systematic review and visualisation*: Yaru Zhou. *Supervision, investigation, writing – review and editing; equal*: Hongyuan Yang. *Data curation, funding acquisition; investigation, methodology, project administration, writing – original draft, writing – review and editing*: Mingming Gao.

## CONFLICT OF INTEREST STATEMENT

The authors declare that they have no conflicts of interest with the contents of this article.

## ETHICS STATEMENT

The experimental design and protocols were approved by the Laboratory Animal Ethical and Welfare Committee of Hebei Medical University (IACUC‐Hebmu‐P 2023031) and were also complied with the Guidelines for Care and Use of Laboratory Animals published by the US National Institutes of Health.

## Supporting information



Supporting Information
