# Supplementary material for: Adipose tissue deficiency impairs transient lipid accumulation and delays liver regeneration following partial hepatectomy in male Seipin knockout mice
Source: Clin Transl Med. 2025 Feb 20;15(2):e70238. doi: 10.1002/ctm2.70238 (PMC11842221; doi:10.1002/ctm2.70238)
Supplement: Supplementary file 1 — Supporting Information [file CTM2-15-e70238-s001.docx]

Adipose Tissue Deficiency Impairs Transient Lipid Accumulation and Delays Liver Regeneration Following Partial Hepatectomy in Male Seipin Knockout Mice

Qianqian Dong, Ziwei Liu, Yidan Ma, Xin Chen, Xiaowei Wang, Jinye Tang, Kexin Ma, Chenxi Liang, Mengyu Wang, Xiaoqin Wu, Yang Liu, Yaru Zhou, Hongyuan Yang, Mingming Gao

**MATERIALS AND METHODS**

**Blood biochemical assay**

Blood samples were collected from the retro-orbital vein of the indicated animals before or at the specified time points after PHx surgery under fed conditions. Plasma levels of triglyceride, total cholesterol, and glucose were measured using the GPO-PAP method, following the manufacturer's guidelines (Bio Sino, Beijing, China). Plasma non-esterified fatty acids (NEFA), β-hydroxybutyrate (β-HB), as well as alanine aminotransferase (ALT) and aspartate aminotransferase (AST), were measured with commercial assay kits (Jiancheng Bioengineering Research Institute, Jiangsu, China). All procedures were carried out in accordance with the manufacturer's instructions.

**Liver lipid extraction**

Liver lipid extraction was performed using a modified Bligh & Dyer method. Approximately 100 mg (wet weight) of frozen liver tissue was homogenized with 1 mL of cold PBS. The liver homogenate was mixed with Folch solution (chloroform = 2:1, v/v) using vortex to extract the lipids. The mixture was then centrifuged at 2,000 rpm for 20 minutes to separate the organic and aqueous phases. The organic phase was evaporated to dryness under a nitrogen stream and dissolved in 500 μL of 3% Triton X-100. Lipid dissolution was facilitated by incubating at 50°C with adequate shaking. Liver triglyceride and total cholesterol levels were determined following the manufacturer's guidelines for the GPO-PAP method (Bio Sino, Beijing, China).

**RNA extraction and Quantitative real-time PCR**

Total RNA was isolated from frozen liver tissue using Trizol reagent (Aidlab Biotechnologies, Beijing, China) following the manufacturer’s protocol. First-strand cDNA was synthesized with a reverse transcription kit (Promega, USA) according to the provided instructions. Quantitative real-time PCR was conducted using 2 × SYBR Green qPCR master mix (Monad, Shanghai, China), with *Gapdh* serving as the reference gene. The primers used for PCR were synthesized by Shanghai Sangon Biotech Company (Shanghai, China) and are listed in Supplementary Table S1.

**Western blotting**

Frozen liver tissue was homogenized in RIPA buffer containing protease inhibitors (Aidlab Biotechnologies, Beijing, China). After centrifugation, the supernatant was collected, and protein concentration was measured using a BCA assay kit (Seven Biotech, Beijing, China). Equal amounts of protein were separated by SDS-PAGE, transferred to a PVDF membrane (Applygen Technologies, Beijing, China), and blocked with 5% milk in TBST for 1 hour. Membranes were incubated with primary antibodies overnight at 4°C, followed by HRP-conjugated secondary antibodies at room temperature for 1.5 hours. Blots were visualized using the ImageQuant LAS 4000 detection system (GE Healthcare, Chicago, IL, USA), and band intensity was quantified with ImageJ software.

The following primary antibodies were used: anti-PCNA (Servicebio, Wuhan, China), anti-AKT and anti-p-AKT (Cell Signaling Technology, Boston, MA, USA), anti-*β*-Actin (Abclonal, Wuhan, China), and Seipin antibody, which was prepared by Proteintech Group Inc and purified by Nieng Yan’s lab at Tsinghua University.

**Hematoxylin and Eosin (H&E) staining**

Mice were anesthetized with 1% sodium pentobarbital and perfused with 0.01M cold PBS. Liver tissues were fixed overnight in 4% paraformaldehyde (PFA) and then trimmed into 5 mm³ pieces. Tissue dehydration process: The liver tissues were sequentially dehydrated by immersion in 85% ethanol at 55°C for 12 minutes, 80% ethanol/20% isopropanol at 55°C for 25 minutes, isopropanol at 55°C for 25 minutes, and paraffin at 75°C for 2 hours before embedding in paraffin using an embedding machine. Sectioning and staining: Paraffin-embedded liver tissues were sectioned into 4-5 μm thick slices, which were then dried and deparaffinized. The sections were rehydrated through a gradient of alcohol and stained with hematoxylin (Baso, Zhuhai, China) and eosin (Solarbio, Beijing, China). After staining, the sections were dehydrated, cleared, and mounted with neutral gum for microscopic examination.

Mitotic Index Calculation: The mitotic index was calculated by counting mitotic hepatocytes in the H&E-stained liver sections. It was expressed as the number of mitotic cells per mm², with five fields examined at 200x magnification for each animal.

**Immunohistochemistry and Immunofluorescence staining**

Paraffin sections of the liver were deparaffinized and rehydrated using the method described for H&E staining. Immunohistochemistry was performed according to the manufacturer’s protocol (Mxb Biotechnologies, Fujian, China) using an anti-PCNA primary antibody (Servicebio, Wuhan, China). Following the immunohistochemical staining, sections were counterstained with hematoxylin, then dehydrated, cleared, and mounted. PCNA-positive nuclei, which appeared brown, were counted in five fields per mouse at 400x magnification. The average number of brown-stained nuclei per field was calculated and presented as PCNA^+^ nuclei/field.

For immunofluorescence staining, liver tissue sections underwent antigen retrieval with citrate buffer, followed by permeabilization with 0.3% Triton X-100. Non-specific binding was blocked using 10% goat serum. The sections were then incubated with a primary Ki67 antibody (Servicebio, Wuhan, China) and a secondary antibody conjugated to TRITC. Nuclei were stained with DAPI. Images were captured using a fluorescence microscope, and Ki67-positive nuclei, appearing red, were quantified at 400x magnification.

**Oil Red O staining**

Fixed liver tissues were trimmed into 5 mm³ blocks and immersed in 20% sucrose solution for 48 hours. The tissue was then embedded in OCT and kept at -20°C. Frozen liver sections, 5-7 µm thick, were stained with Oil Red O at 37°C for 30 minutes. After staining, sections were counterstained with hematoxylin to visualize the nuclei. The sections were mounted with 90% glycerol and examined under a microscope to analyze lipid deposition.

**Supplementary Figures**


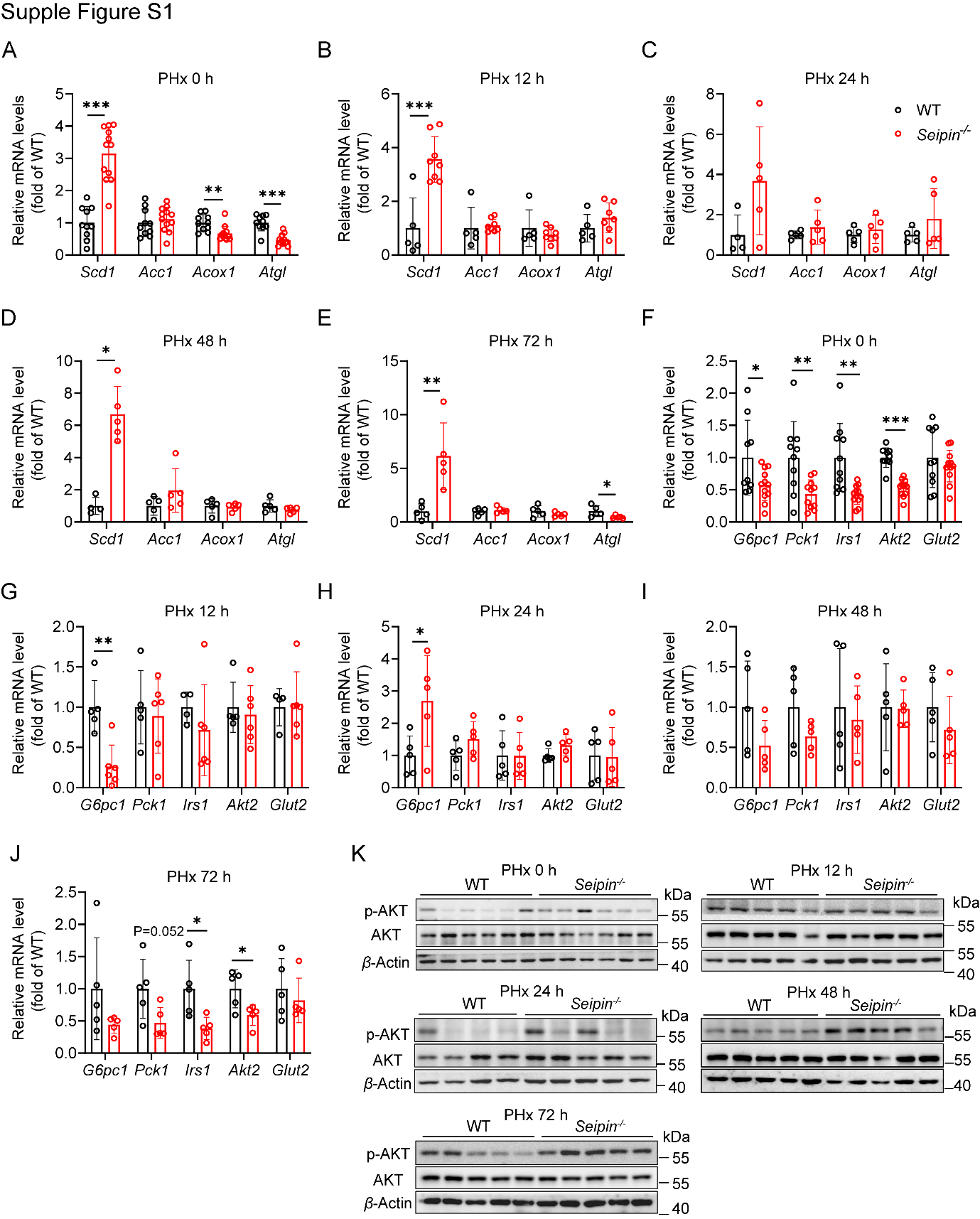


**Figure S1. Time-Dependent Changes in Lipid Metabolism and Insulin Signaling in the Liver of WT and *Seipin^-/-^* Mice Post-PHx.**

A-E. Relative mRNA expression levels of lipid metabolism genes in the liver of WT and *Seipin^-/-^* mice at 0 h (A), 12 h (B), 24 h (C), 48 h (D), and 72 h (E) post-PHx (n = 4-12). Data are presented as mean ± SD. Statistical significance was determined by two-tailed Student’s *t*-test. **p* < 0.05, ***p* < 0.01, ****p* < 0.001.

F-J. Relative mRNA expression levels of insulin signaling pathway genes in the liver of WT and *Seipin^-/-^* mice at 0 h (F), 12 h (G), 24 h (H), 48 h (I), and 72 h (J) post-PHx (n = 5-12). Data are presented as mean ± SD. Statistical significance was determined by two-tailed Student’s *t*-test. **p* < 0.05, ***p* < 0.01.

K. Western blot analysis of pAKT and AKT protein levels in the liver of WT and *Seipin^-/-^* mice at 0 h, 12 h, 24 h, 48 h, and 72 h post-PHx (n = 4-6).


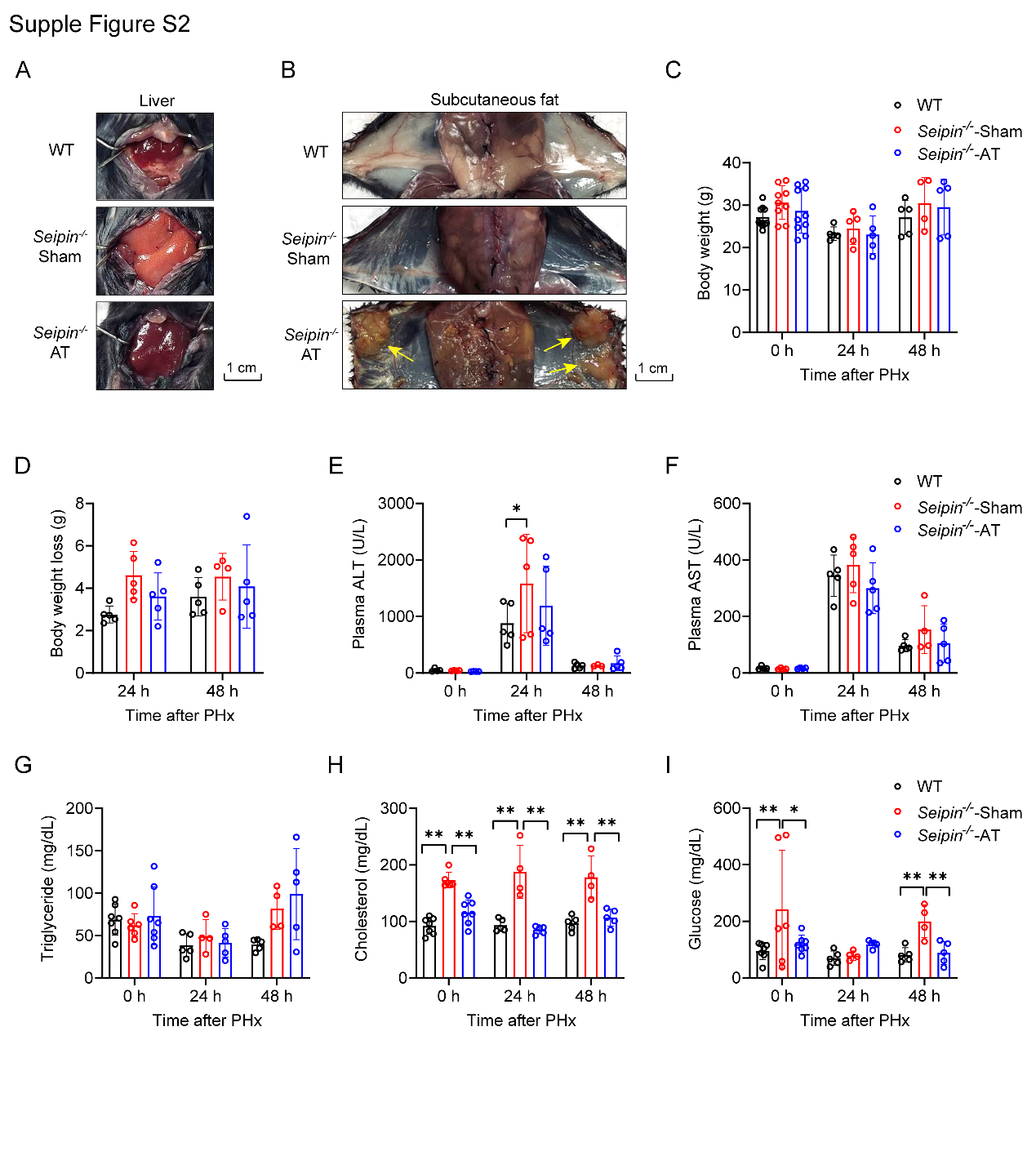


**Figure S2. Physiological and biochemical analysis of WT, *Seipin^-/-^*-Sham, and *Seipin^-/-^*-AT mice post-PHx.**

A. Representative images of liver tissue from WT (upper), *Seipin^-/-^*-Sham (middle), and *Seipin^-/-^*-AT (lower) mice after 12 weeks of adipose tissue transplantation. Scale bar = 1 cm.

B. Representative images of subcutaneous fat in WT (upper), *Seipin^-/-^*-Sham (middle), and *Seipin^-/-^*-AT (lower) mice at the time of sacrifice. The yellow arrow indicates the transplanted fat grafts in *Seipin^-/-^*-AT mice. Scale bar = 1 cm.

C. Body weight of WT, *Seipin^-/-^*-Sham, and *Seipin^-/-^*-AT mice at 0 h, 24 h, and 48 h post-PHx (n = 4-10). Data are presented as mean ± SD.

D. Body weight loss of WT, *Seipin^-/-^*-Sham, and *Seipin^-/-^*-AT mice at 24 h, and 48 h post-PHx, calculated as the reduction in body weight compared to pre-PHx levels (n = 4-5). Data are presented as mean ± SD.

E-F. Plasma ALT (E) and AST (F) levels in WT, *Seipin^-/-^*-Sham, and *Seipin^-/-^*-AT mice at 0 h, 24 h, and 48 h post-PHx (n = 4-5). Data are presented as mean ± SD. Statistical significance was determined by two-way ANOVA with Tukey's multiple comparison test. **p* < 0.05.
G-I. Plasma triglycerides (G), total cholesterol (H), and glucose (I) levels in WT, *Seipin^-/-^*-Sham, and *Seipin^-/-^*-AT mice at 0 h, 24 h, and 48 h post-PHx (n = 4-7). Data are presented as mean ± SD. Statistical significance was determined by two-way ANOVA with Sidak's multiple comparison test. **p* < 0.05, ***p* < 0.01.


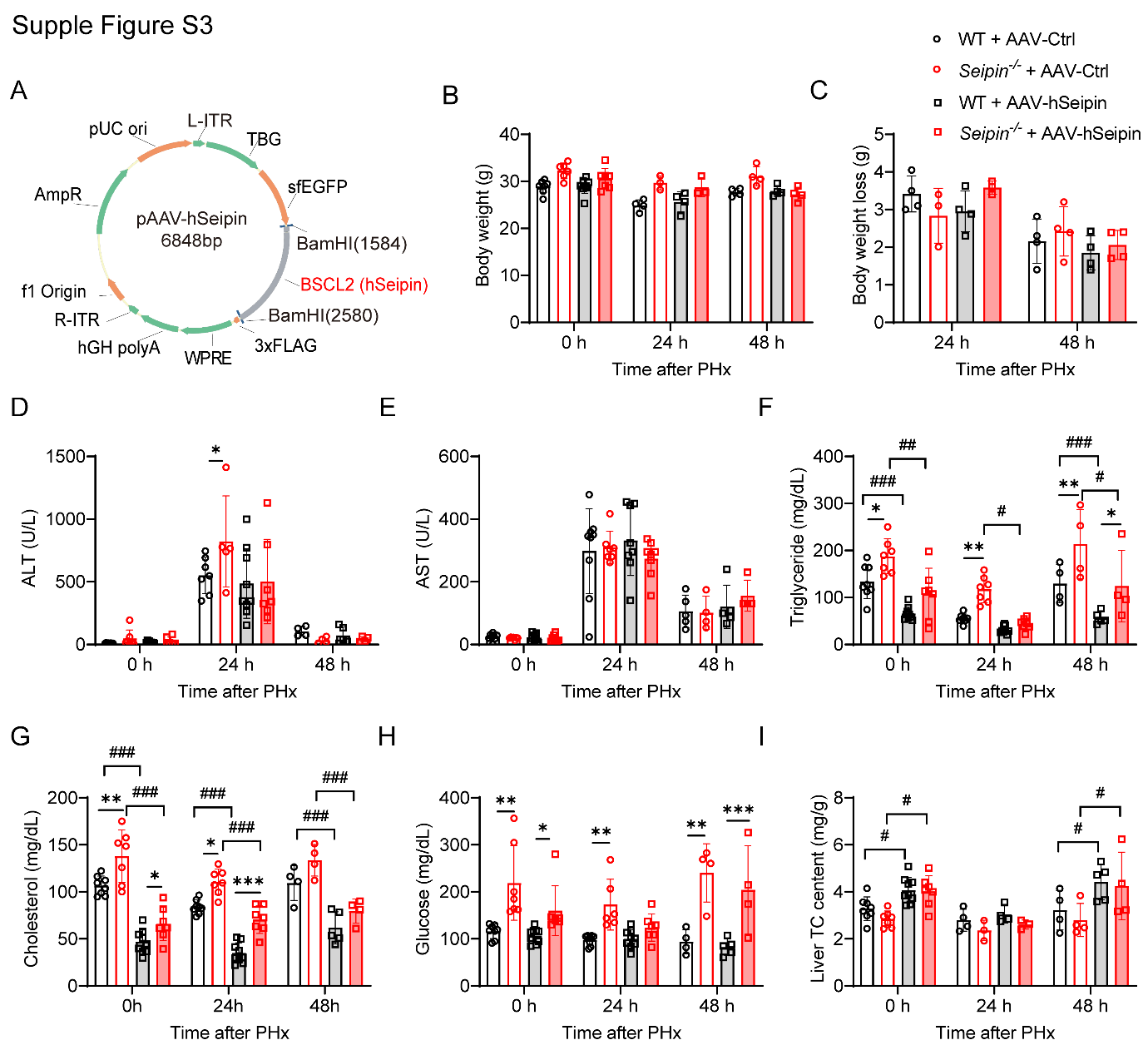


**Figure S3. Physiological and biochemical analysis of AAV-Ctrl and AAV-hSeipin injected mice post-PHx.**

A. Full map of adeno-associated virus after hSeipin gene insertion, with the human Seipin gene length of 1263 bp.

B. Body weight of WT + AAV-Ctrl, *Seipin^-/-^* + AAV-Ctrl, WT + AAV-hSeipin and *Seipin^-/-^* + AAV-hSeipin mice at 0 h, 24 h, and 48 h post-PHx (n = 3-9). Data are presented as mean ± SD.

C. Body weight loss of WT + AAV-Ctrl, *Seipin^-/-^* + AAV-Ctrl, WT + AAV-hSeipin and *Seipin^-/-^* + AAV-hSeipin mice at 0 h, 24 h, and 48 h post-PHx after PHx, calculated as the reduction in body weight compared to pre-PHx levels (n = 3-4). Data are presented as mean ± SD.

D-E. Plasma ALT (D) and AST (E) levels in WT + AAV-Ctrl, *Seipin^-/-^* + AAV-Ctrl, WT + AAV-hSeipin and *Seipin^-/-^* + AAV-hSeipin mice at 0 h, 24 h, and 48 h post-PHx (n = 4-9). Data are presented as mean ± SD. Statistical significance was determined by two-way ANOVA with Sidak's multiple comparison test. **p* < 0.05.

F-H. Plasma triglycerides (F), total cholesterol (G), and glucose (H) levels in WT + AAV-Ctrl, *Seipin^-/-^* + AAV-Ctrl, WT + AAV-hSeipin and *Seipin^-/-^* + AAV-hSeipin mice at 0 h, 24 h, and 48 h post-PHx, measured using enzymatic kits (n = 4-9). Data are presented as mean ± SD. Statistical significance was determined by two-way ANOVA with Sidak's multiple comparison test. **p* < 0.05, ***p* < 0.01, ****p* < 0.001 (WT vs. *Seipin^-/-^*), ^#^*p* <0.05, ^##^*p* <0.01, ^###^*p* <0.001 (AAV-Ctrl vs. AAV-hSeipin).

I. Liver total cholesterol levels in WT + AAV-Ctrl, *Seipin^-/-^* + AAV-Ctrl, WT + AAV-hSeipin and *Seipin^-/-^* + AAV-hSeipin mice at 0 h, 24 h, and 48 h post-PHx, measured using enzymatic kits (n = 3-9). Data are presented as mean ± SD. Statistical significance was determined by two-way ANOVA with Sidak's multiple comparison test. ^#^*p* <0.05 (AAV-Ctrl vs. AAV-hSeipin).

**Supplementary Table S1. Real time PCR primer information.**

| Gene | Sense strand (5′-3′) | Antisense strand (5′-3′) |
| --- | --- | --- |
| hSeipin  Cd36  Fabp4 | ACTTCTTCGCGTTCGGTGATGC  GGCAGGAGTGCTGGATTA  AAGGTGAAGAGCATCATAACCCT | CTGGATGCGCTTGCTGTGGAT  GAGGCGGGCATAGTATCA  TCACGCCTTTCATAACACATTCC |
| Scd1 | TGACCTGAAAGCCGAGAA | ATGTGCCAGCGGTACTCA |
| Acc1 | CTCCCGATTCATAATTGGGTCTG | TCGACCTTGTTTTACTAGGTGC |
| Acox1 | GTACCAGCGTCGGGGATTG | AAAGGCTCAGGATGCCCTCG |
| Atgl | TGCAGCACATTTATCCCGGT | GCAAAGGGTTGGGTTGGTTC |
| G6pc1 | AATCTCCTCTGGGTGGCA | GCTGTAGTAGTCGGTGTCC |
| Pck1 | TGCGGATCATGACTCGGATG | AGGCCCAGTTGTTGACCAAA |
| Irs1 | GGGACTGGGGGAGACATAGT | GGCAATGGCAAAGTGTTCGT |
| Akt2 | GGCCCCTGACCAGACCTTA | GATAGCCCGCATCCACTCTTC |
| Glut2 | GCCCAGCAGTTCTCAGGAAT | ACATGCCAATCATCCCGGTT |
| Ccnd1 | TCAAGTGTGACCCGGACTGC | CCTTGGGGTCGACGTTCTG |
| *Gapdh* | TGATGACATCAAGAAGGTGGTGAAG | TCCTTGGAGGCCATGTAGGCCAT |

**Supplementary Table S2. Key resources table.**

| REAGENT or RESOURCE | SOURCE | IDENTIFIER |
| --- | --- | --- |
| Antibodies |  |  |
| Rabbit polyclonal anti-PCNA | Servicebio | Cat#GB11010;  RRID: AB_2811188 |
| Rabbit polyclonal anti-Ki67 | Servicebio | Cat#GB111499  RRID: AB_2927572 |
| Rabbit monoclonal anti-AKT | Cell Signaling Technology | Cat#4691;  RRID: AB_915783 |
| Rabbit monoclonal anti-p-AKT | Cell Signaling Technology | Cat#4060;  RRID: AB_2315049 |
| Rabbit monoclonal anti-Seipin | This lab | N/A |
| HRP conjugated Goat-anti-Rabbit IgG | Servicebio | Cat#GB23303;  RRID: AB_2811189 |
| Rabbit monoclonal anti-*β*-actin | Abclonal | Cat#AC026;  RRID: AB_2768234 |
| Chemicals |  |  |
| Total cholesterol assay kit | Biosino | CAT#100000180 |
| Triglyceride assay kit | Biosino | CAT#100000220 |
| Glucose assay kit | Biosino | CAT#100000240 |
| β-HB assay kit | Jiancheng | CAT# E030-1-1 |
| NEFA assay kit | Jiancheng | CAT#A042-2-1 |
| ALT/GPT Kit | Jiancheng | CAT#C009-2-1 |
| AST/GOT Kit | Sigma | CAT#C010-2-1 |
| Hematoxylin | Baso | CAT#BA4097 |
| Eosin | Solarbio | CAT#G1100 |
| Oil Red O | Sigma | CAT#1320-06-5 |
| UltraSensitive^TM^ SP IHC Kit | MXB Biotechnologies | CAT# KIT-9710 |
| BCA Protein Assay Kit | Seven | CAT#SW101 |
| 30% PAGE | Servicebio | CAT#G2004 |
| Protein maker | Thermo Fisher | CAT#26616 |
| ECL kit | Servicebio | CAT#G2014 |
| Triton X-100 | Solarbio | CAT#T8200 |
| Protease inhibitor | Servicebio | CAT#G2006 |
| SweScript All-in-One RT SuperMix | Servicebio | CAT#G3373 |
| TRIzol Reagent | TIANGEN | CAT#DP424 |
| 2×SYBR Green qPCR master Mix | Servicebio | CAT#G3326 |
| SDS | Coolaber | CAT#CS9701 |
| TEMED | Sigma | CAT#T8090 |
| Experimental models and virus strains | | |
| *Seipin^-/-^* mice  AAV8-TBG-GFP | This lab  OBIO | N/A  N/A |
| AAV8-TBG-hSeipin | OBIO | N/A |
| Software and algorithms |  |  |
| ImageJ | National Institutes of Health | https://ImageJ.net/software/ImageJ |
| GraphPad Prism 9.0 | GraphPad | https://www.graphpad.com |
| Adobe Photoshop CS6 | Adobe | https://www.adobe.com |
